# Locomotion and disaggregation control of paramagnetic nanoclusters using wireless electromagnetic fields for enhanced targeted drug delivery

**DOI:** 10.1038/s41598-021-94446-4

**Published:** 2021-07-23

**Authors:** Kim Tien Nguyen, Gwangjun Go, Jin Zhen, Manh Cuong Hoang, Byungjeon Kang, Eunpyo Choi, Jong-Oh Park, Chang-Sei Kim

**Affiliations:** 1 Korea Institute of Medical Microrobotics (KIMIRo), 43-26 Cheomdangwagi-ro, Buk-gu, Gwangju, Korea; 2 School of Mechanical Engineering, Chonnam National University, 77 Yongbong-ro, Buk-gu, Gwangju, Korea; 3 College of AI Convergence, Chonnam National University, 77 Yongbong-ro, Buk-gu, Gwangju, Korea; 4 College of Medical Engineering, Xinxiang Medical University, Xinxiang, Henan China

**Keywords:** Techniques and instrumentation, Electrical and electronic engineering, Biomedical engineering

## Abstract

Magnetic nanorobots (MNRs) based on paramagnetic nanoparticles/nanoclusters for the targeted therapeutics of anticancer drugs have been highlighted for their efficiency potential. Controlling the locomotion of the MNRs is a key challenge for effective delivery to the target legions. Here, we present a method for controlling paramagnetic nanoclusters through enhanced tumbling and disaggregation motions with a combination of rotating field and gradient field generated by external electromagnets. The mechanism is carried out via an electromagnetic actuation system capable of generating MNR motions with five degrees of freedom in a spherical workspace without singularity. The nanocluster swarm structures can successfully pass through channels to the target region where they can disaggregate. The results show significantly faster response and higher targeting rate by using rotating magnetic and gradient fields. The mean velocities of the enhanced tumbling motion are twice those of the conventional tumbling motion and approximately 130% higher than the gradient pulling motion. The effects of each fundamental factor on the locomotion are investigated for further MNR applications. The locomotion speed of the MNR could be predicted by the proposed mathematical model and agrees well with experimental results. The high access rate and disaggregation performance insights the potentials for targeted drug delivery application.

## Introduction

Over the past decade, numerous untethered externally powered microrobots and nanorobots have been developed for biomedical applications^1–8^. Wirelessly powered microrobots/nanorobots have been shown to have potential, particularly in targeted cancer therapy, as they offer advantages in performing tasks in minimally invasive surgery, including targeting inaccessible parts of the human body^9–12^. Targeted drug delivery can remarkably improve the access rate of drugs to the target, increase drug absorption, and minimize the required dose. It can also reduce damage to healthy cells compared to conventional therapy methods, such as chemotherapy, where more than 99% of the drug eventually ends up in normal cells^13^.

To successfully control untethered microrobots/nanorobots in the human body, a number of critical problems should be solved. The first problem is to find a way to transmit the power required for robot motion through human tissues. Among the external power sources, the electromagnetic field has been considered as a distinct solution, because it can penetrate the human body and has been shown to be compatible with medical use^14^. With its help, microrobots/nanorobots can be controlled to reach their targets through the interaction of magnetic force and torque. Second, the physics governing microrobot/nanorobot motion is greatly different from the motion in macro-scale environment, where surface friction, Van der Waals, and electrostatic forces dominate inertial forces^15^. Furthermore, microrobots/ nanorobots are expected to move in the bloodstream, where blood is considered as an inhomogeneous, non-Newtonian fluid; blood flow and wall effects strongly interfere with robot motion. Consequently, the control of untethered microrobots/nanorobots in targeted drug delivery using an electromagnetic field is limited by several factors, such as the structure, material, and locomotion method of microrobot/nanorobot, as well as the achievable strengths of the magnetic and gradient fields of the actuation system. Motivated by naturally inspired mechanisms, effective control of robot locomotion is researched to improve robot performance. Eukaryotic flagellum or sperm-inspired microrobots driven by wave propulsion using an oscillating field probably have the most efficient motion in fluids with a low Reynolds number^16,17^. Helical propulsion of bacterial flagellum-inspired microrobots also shows the very accurate performance when using a rotating magnetic field^18,19^. However, in a targeting task, a robot must carry the drug cargo through blood vessels that are only a few micrometers in diameter. The reduction of the structure size to a few micrometers and the amount of payload dose carried by microrobots were limited^3^. Therefore, the development of controllable nano-agents is required to carry the drug or magnetic nanoparticle itself as a therapeutic agent. In particular, the dispersion of nanorobots in bio-fluids immediately after injection is typically large; therefore, individual control of these robots by induction of extremely low magnetic forces must be addressed.

One possible solution to the above problems is to aggregate the nanorobots into chains or clusters so that they can move in unison, allowing the total magnetic torque and force acting on the aggregated structures to be sufficient for locomotion. In addition to clustering, these structures must be able to disaggregate after reaching the target or entering a narrow vessel. Snezhko et al. presented an extremely interesting cluster of self-assembled colloidal asters based on magnetic nanoparticles by applying a rotating field to the nanoparticle suspension confined between two immiscible liquids. The aster-like swarm can be controlled to change shape by changing frequency; it is also capable of moving and can perform the grasping function using the directed in-plane field^20^. However, the application of this approach to a real in vivo environment in blood vessels is limited because the assembly principle only occurs at the interface of two immiscible fluids. Yu et al. and Mohorič et al. reported that rotating nanoparticle chains could be induced to swarm out into a vortex-like structure as well as perform translational motion and pick-and-place function using the in-plane rotating magnetic field by changing the pitch angle^21–24^. By changing the rotation frequency, this approach was able to generate a single vortex or multiple vortices. Recently, the ribbon-like swarm has shown promising locomotion, capable of size elongation, dividing, merging, and even retaining shape when it encounters a boundary^25,26^. This method has been studied in channels containing various bio-fluids. Xie et al. demonstrated rapid and reversible transformation between liquid, chain, vortex, and ribbon-like swarms to quickly adapt to different environmental boundaries and perform various tasks^27^. These microrobotic swarms have relatively strong dipole–dipole interactions that help maintain the pattern during locomotion. However, these swarm control require a strictly static environment and a certain amount of time (tens of seconds) for self-assembly, which is practically not applicable for the drug delivery system for an intravascular environment. From the clinical perspective of intravascular drug delivery, the environment is highly dynamic with intense and blood flow. On the other hand, although the gradient pulling of magnetic particles has a rather weak dipole–dipole interaction, it has exhibited considerable ability to deliver a drug to the tumor site in an in-vivo experiment^28,29^. Tumbling only uses the magnetic field to rotate perpendicular to the working plane, which results in an effective locomotion speed^30–32^.

**Figure 1:**
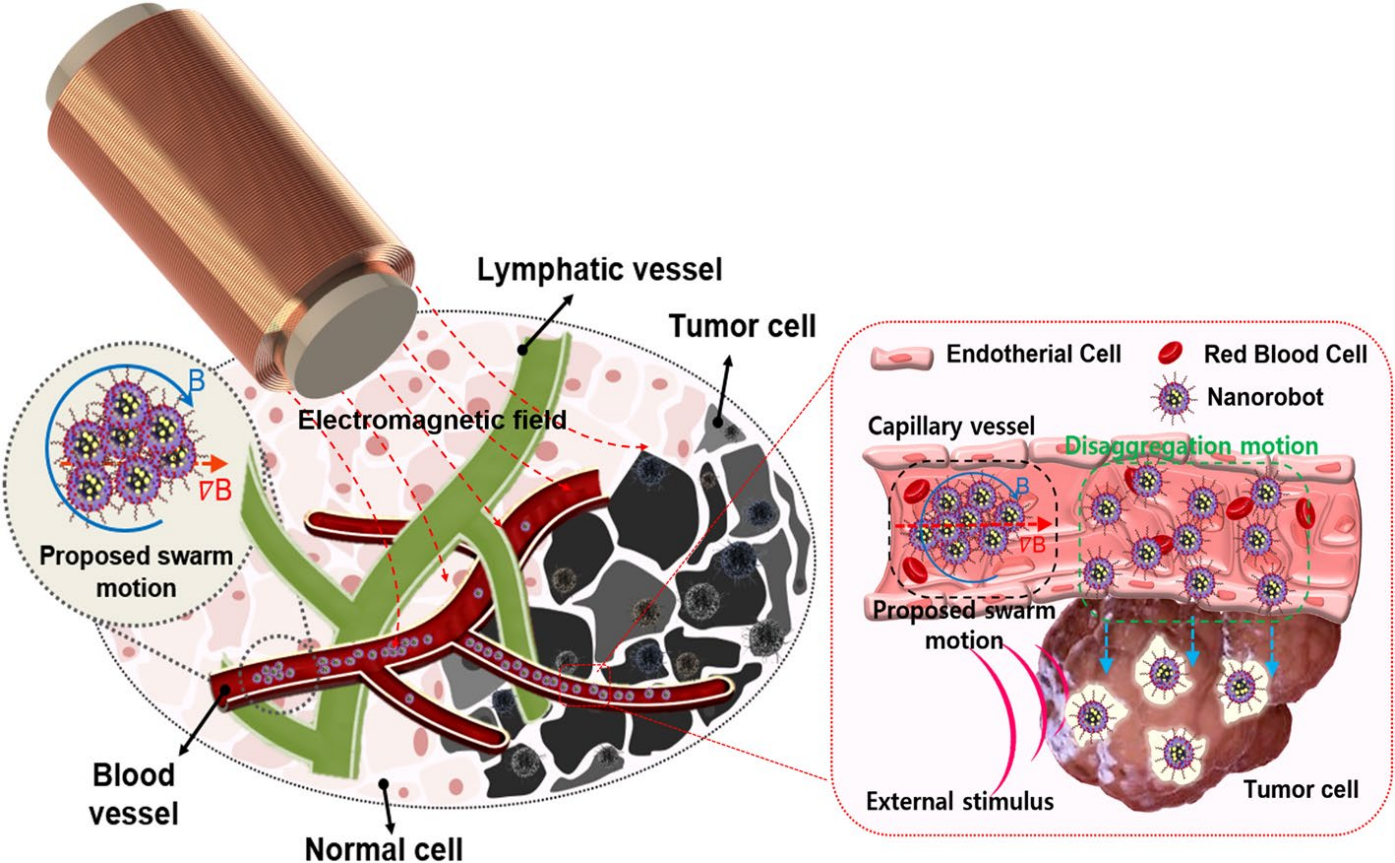
Conceptual schematic of targeted drug delivery. Nanorobots are driven to targeted area by EnEMAs with proposed swarm motion and visualized by X-ray monitoring; enhanced tumbling motion steers magnetic clusters to reach target area, and disaggregation motion breaks cluster into smaller sizes to enter micro-vessels at site. Drug release is triggered by external stimulus after approaching target area.

In this work, a new approach to manipulate magnetic nanoparticles to improve the locomotion and access rate for targeted drug delivery is proposed. Technical differences distinguished from the already existing methods are summarized in Table 1. The concept of targeted drug delivery using paramagnetic nanoparticles as therapeutic nano-agents is shown in Fig. 1. The enhanced tumbling motion induced by the gradient and rotating magnetic fields are implemented in the experimental results and quantified by the analysis. The desired rotating field and gradient are generated by a proposed new magnetic manipulation system called Ennead Electromagnets Actuation system (EnEMAs), which is capable of five degrees of freedom (DOF) to control magnetic objects in a large spherical working space without singularity.

**Table 1:** Comparison with the existing microrobotic swarm motions.

Chain-like: Rolling	Chain-like: Tumbling	Chain-like: Pulling	Chain-like: Proposed
			
2D rotating field on demanded plane^27,31,32^	2D rotating field on demanded plane^27,30^	Static field and gradient on XY plane^28,29^	2D rotating field on plane with gradient field along with desired direction
- Static env - Free pattern	- Static env - Free pattern	- Static and dynamic env - Free pattern	- Static and dynamic env - Free pattern

Vortex-like		Ribbon-like	Aster-like
			
2D rotating field on demanded plane^21–23^	3D rotating field^24^	2D oscillating field^25,26^	2D oscillating field on demanded plane^20^
- Static env - Stable pattern - Controllable pattern’s size and shape		- Static env - Stable pattern - Controllable pattern	- Static env - Stable pattern - Controllable pattern

The proposed method involves a novel locomotion method for magnetic nanoparticles (MNPs), called the enhanced tumbling, which can improve the performance of MNPs. In addition, quantitative analysis through simulation and experiments was performed to design the MNP delivery system in terms of gradient field strength, rotating field frequency, and cluster size to improve the cluster locomotion performance. By maximizing the advantages of both gradient and rotating fields controlled by the EnEMAs, the use of the proposed method leads to faster movement and access rate of the MNP clusters to reach a target region.

## Materials and methods

### Synthesis of nanoclusters

**Figure 2:**
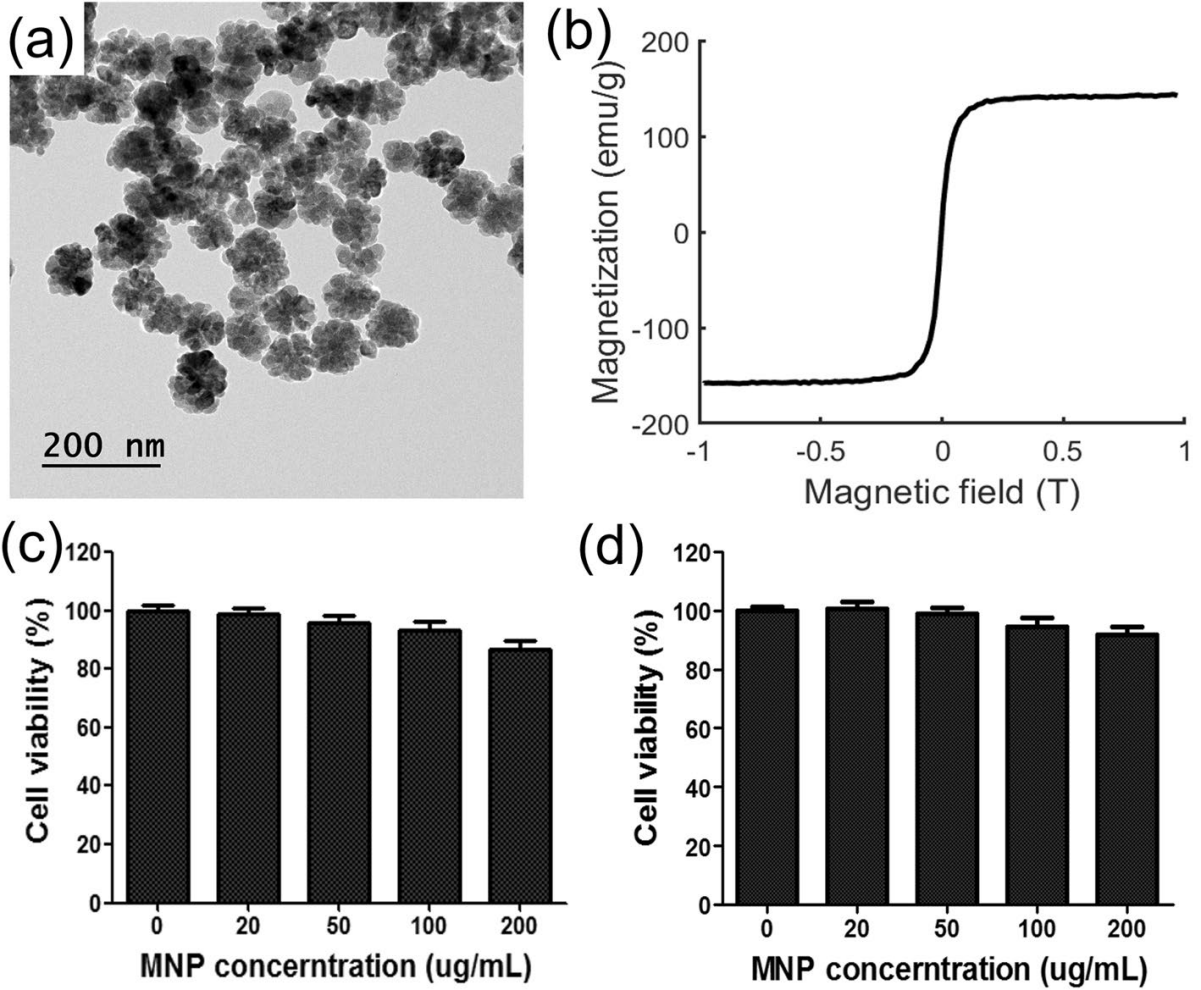
Characteristic and biocompatibility of nanoparticles used for locomotion method. (**a**) TEM image of nanoparticles, (**b**) magnetization curve of fabricated nanoparticles obtained using vibrating sample magnetometer, (**c**) cell viability on NIH3T3, and (**d**) cell viability on 4T1 cells.

Magnetic nanoclusters (MNRs) were prepared according to the solvothermal method published in literature with slight modifications^33^, as reported in our previous work^28^. In a mixture of ethylene glycol (EG) and diethylene glycol (DEG) (EG:DEG = 1:4; total volume: 20 mL), FeCl3.6H2O (2 mmol) was dissolved in a beaker under magnetic stirring. After 30 min, 1.5 g of polyvinylpyrrolidone was added to this solution and then heated at 120 °C for 1 h to obtain a transparent solution. Then, 1.5 g of sodium acetate (NaOAc) was added into the solution and vigorously stirred for 30 min. The resulting homogeneous solution was transferred into a Teflon-lined stainless-steel autoclave and then heated at 200 °C. After a 12 h-reaction process, the autoclave was cooled to room temperature, and the obtained MNR was washed with ethanol and water three times. The produced black nanoclusters were further modified using polyethyleneimine (PEI) mixed with PEI (0.5 g/mL) and heated at 60 °C for 3 h. The product was again rinsed with water three times, and magnetic separation was executed to remove extra molecules. Figure 2a,b show the transmission electron microscopy (TEM) images of the fabricated nanoclusters with an average diameter of 98 nm.

### Biocompatibility of MNR

The biocompatibility of MNR was investigated on 4T1 (mice breast cancer cell line) and NIH3T3 (mice fibrous cell line) cells. The cells were seeded in a 96-well plate at a density of 1 × 104 cells/well and incubated overnight for cell attachment. Thereafter, the cells were treated with various MNR concentrations (0–200 μg/mL) and further incubated for 24 h. After the incubation, 25 μL of MTT solution (5 mg/mL in phosphate-buffered saline (PBS)) was added to each well and placed in the incubator for another 4 h. Then, the MTT solution was aspirated, and 100 μL of DMSO was added to dissolve the cells for spectrum measurement. Finally, the cell viability was measured by a microplate reader at an absorbance of 570 nm (Thermo Scientific, Waltham CA). Figure 2c,d show the cell viability of MNR on the NIH3T3 and 4T1 cells, respectively. These results indicate considerable biocompatibility on the NIH3T3 and 4T1 cells (more than 90% in all cases) in which the cell viability decreased with the increase in the MNR concentration (0–200 μg/mL).

### Electromagnetic actuation system

**Figure 3:**
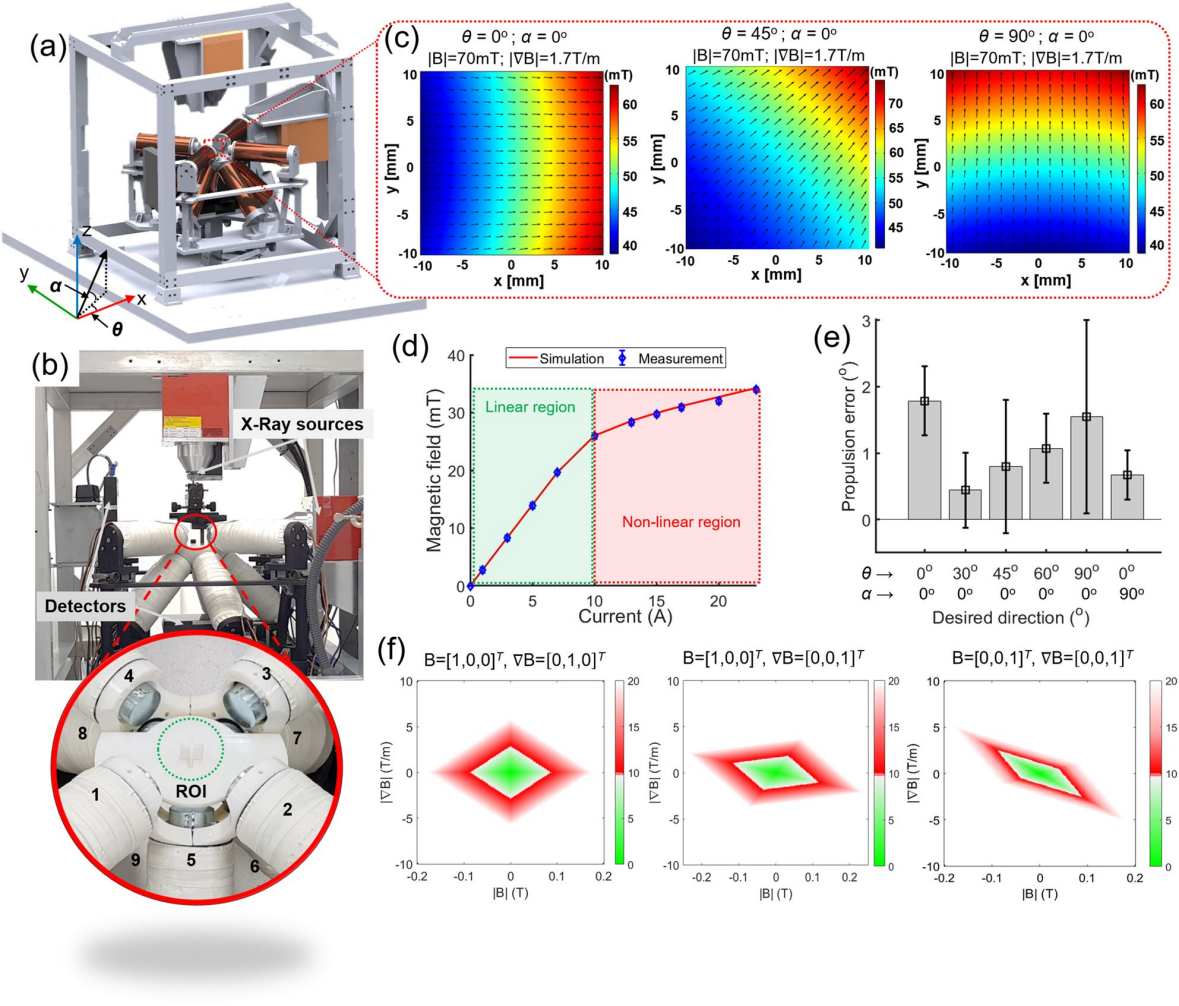
Design and implementation of EMA system. (**a**) Design of enhanced EMA system integrated with biplane X-ray system. θ and α are the alignment angle of the object’s desired orientation projected onto the X axis and XY plane, respectively. (**b**) Digital photograph of EMA system used in this work for investigating nanorobot motion. (**c**) COMSOL simulation results of magnetic field map generated by EnEMA system with magnetic and gradient fields of 70 mT and 1.7 T/m at orientations of *θ* = 0°, 45°, 90° and *α* = 0°, respectively. (**d**) Measured and simulated B–I curves of electromagnetic coil showing linearity of generated field with respect to applied current; green area represents linear region in which generated field is highly linear with respect to applied current; red area indicates non-linear region due to core saturation. (**e**) Propulsion test of system using 300 µm cylindrical magnet at 5 mT and 0.1 T/m. (**f**) Achievable magnetic and gradient fields in workspace at three orientations: θ_*B*_ = 0°, α_*B*_ = 0°, θ_*F*_ = 90°, α_*F*_ = 0°; θ_*B*_ = 0°, α_*B*_ = 0°, θ_*F*_ = 0°, α_*F*_ = 90°; θ_*B*_ = 0°, α_*B*_ = 90°, θ_*F*_ = 0°, α_*F*_ = 90°. In which *θ*_*B*_ and *θ*_*F*_ are the magnetic field and magnetic force angle along with the *θ* direction, respectively, and *α*_*B*_ and *α*_*F*_ are the magnetic field and magnetic force angle along with the *α* direction, respectively.

The MNR control task involving the use of both magnetic field and field gradient to control the MNR locomotion requires both heading and force control. Therefore, the total number of magnetic sources should be equal to or greater than eight^34^. In this work, a total of nine magnetic sources are employed to generate strong magnetic and field gradients for the study of the proposed magnetic particles locomotion, i.e., the EnEMAs configured with multiple soft-core electromagnetic coils. The soft-core material used in this work is pure iron (DT4C)^35^, and the system was designed for in vivo experiments on small mammals, such as rats. The minimum distance from each coil to the center was set at 60 mm. The use of the biplane X-ray system as the visual feedback in the targeting process is anticipated in future in vivo experiments; therefore, the top and side views should be maintained for visualization purposes. With these design constraints, the configuration of the proposed EMA system was set with four in-plane upper coils and five tilted coils at the bottom (Fig. 3a,b). The range of angles for coil optimization with respect to the above design constraints is shown in Fig. S1 and Table S2. The system was optimized using an algorithm (details can be found in “Introduction” section of the supplementary material). The design parameters of the EnEMAs are summarized in Table S1. The results of the optimized coil configuration are shown in Fig.S2 and Table S3.

In order to characterize the system, the model of the proposed magnetic system was formulated by finite element method and implemented in COMSOL Multiphysics 5.3, Inc. MA, USA. In Fig. 3c, the magnetic and gradient fields of 70 mT and 1.7 T/m are simulated with orientations of θ = 0°, 45°, 90°, and α = 0°, respectively. The magnetic field at the center of the workspace measured with the three-axis Tesla meter (8030, F. W. Bell, USA), as shown in Fig. 3d and summarized in Table S4, is compared with the simulation results. In this field, the cores can linearly generate the magnetic flux density with a current of up to 10 A. Finally, the open-loop alignment test with the calibrated system manipulates the 300 μm cylindrical magnet in an acrylic container filled with high-viscosity silicone oil (350 cP). The purpose of this experiment is to evaluate the accuracy of the magnetic field and force generated by the calibrated system in terms of alignment and uniformity. For an accurate evaluation of the system, the control object must have a constant magnetization orientation and amplitude and be highly sensitive to the orientation of the magnetic field. Therefore, the permanent magnet is used for this propulsion test, where the generated field orientation can be easily interpreted from its motion. In Fig. 3e, the magnet was pulled in different directions (θ = 0°, 30°, 45°, 60°, 90°; α = 0°, 90°) with a mean error of 1.05° under constant magnetic and gradient fields of 5 mT and 0.1 T/m, respectively. Figure 3f shows the achievable magnetic and gradient fields in the workspace. With nine electromagnetic coils, the EnEMAs is capable of 5-DOF heading and force control with maximum generated magnetic and gradient fields of 180 mT and 5 T/m. These fields are generated simultaneously with a maximum applied current of 20 A, in which the current outside the linear range (10 A) is compensated based on the B–I curve shown in Fig. 3d.

### Enhanced tumbling motion of nanocluster

The tumbling motion has shown to be efficient in manipulating microparticle/nanoparticle chains by utilizing the rotating magnetic field^36^. In fluids with a low Reynolds number, the reciprocal motion of symmetric objects leads to zero net translation motion if the fluid is Newtonian and incompressible^15^. In tumbling motion, this scallop theorem can be broken by the interaction with viscous friction in nearby surfaces, assuming that the surfaces involved in this motion have wide areas. This is a major problem in the pulling motion of microparticle/nanoparticle chains using gradient field, despite the 100% swimming efficiency with gradient pulling^9,37,38^. In this work, an enhanced tumbling motion with fast locomotion speed and high targeting accuracy is proposed, which is sufficient for targeted drug delivery with a high access rate.

**Figure 4:**
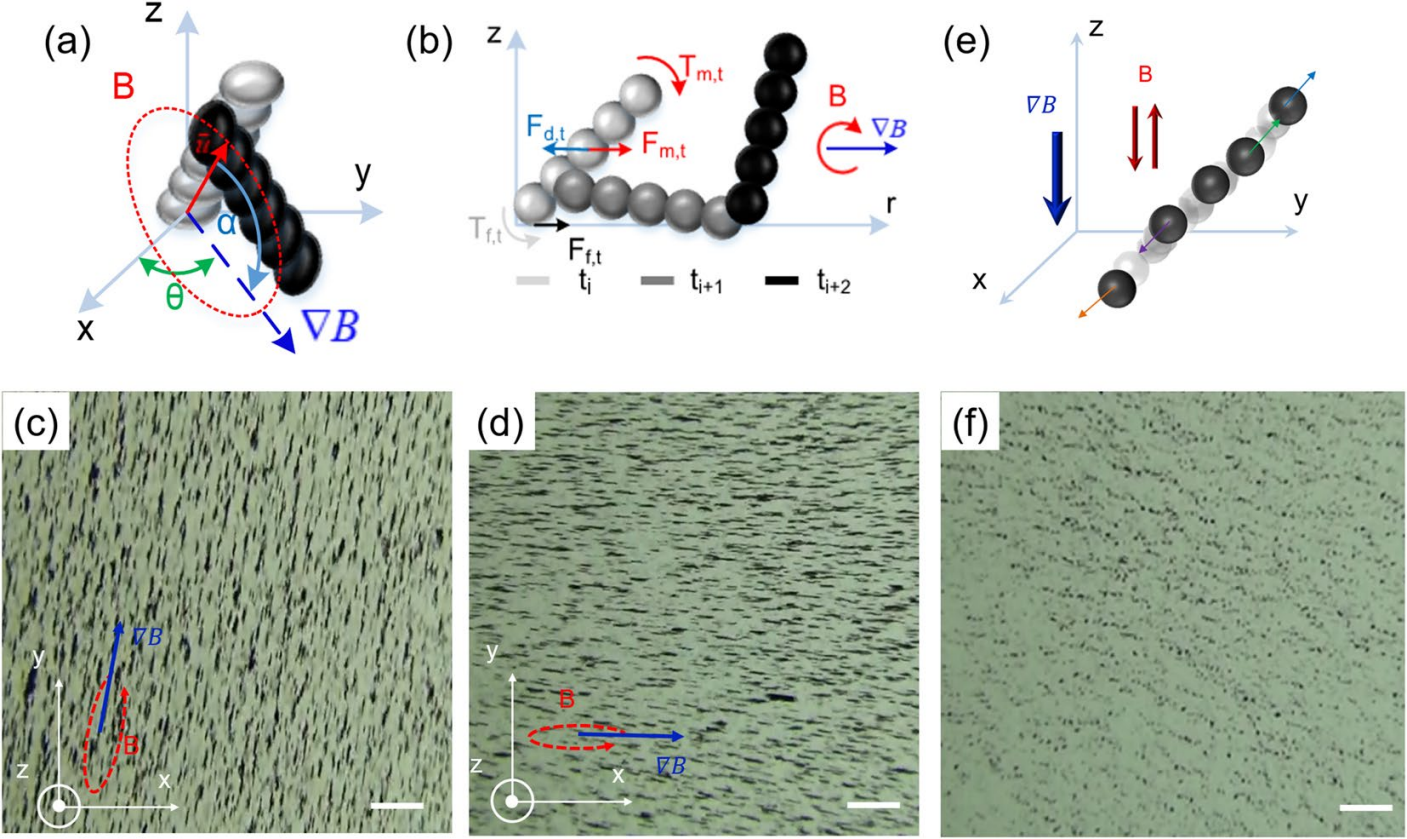
Enhanced tumbling motion of nanoparticles. (**a**) Three-dimensional schematic of magnetic field and gradient field applied to generate enhanced tumbling motion. (**b**) Two-dimensional force and torque exerted on particle chains in proposed locomotion method. (**c,d**) Captured images of particles chains using proposed method in two different control orientations. (**e**) Disaggregation mechanism of particle chain. (**f**) Combination of perpendicular alternative field and gradient field breaks chain structure by chain–chain and dipole–dipole repulsion as illustrated by captured image (**f**). Scale bars: 500 µm.

The magnetic nanoparticle chains have preferential magnetization along their axes. To achieve enhanced tumbling motion, a magnetic field is first applied to the nanoparticle chains. This results in a magnetic torque that can align the chains along the direction $\vec{u}$, as shown in Fig. 4a.

1 $$ \vec{u} = \left[ {\begin{array}{*{20}c} {\cos (\alpha )\cos (\theta )} & {\cos (\alpha )\sin (\theta )} & {\sin (\alpha )} \\ \end{array} } \right] $$

By turning the magnetic field in a plane perpendicular to the working plane, the desired rotating magnetic field in three-dimensional space is expressed in a parametric equation as follows:

2 $$ B = B_{o} \left( {\cos (\omega t)\vec{u} + \sin (\omega t)\vec{n} \times \vec{u}} \right) $$

where *B*_*o*_, $\omega$, $\vec{n}$ and are the magnitude, the angular velocity of the desired external electromagnetic field, and the unit normal vector of the desired electromagnetic field plane, respectively. The magnetic gradient is then applied to the working plane according to the desired in-plane propulsion angle, *θ*. To clarify the enhanced tumbling motion mechanism, the force and torque balance in the rotational directions are considered (Fig. 4b). First, the forces in the y-direction in which the inertia term is neglected are summed as:

3 $$ \sum F = F_{m} + F_{f} - F_{d} = 0 $$

where the magnetic force, *F*_*m*_, friction force, *F*_*f*_^39^, and drag force^30^, *F*_*d*_, acting on the nanoparticle chains are expressed as follows:

4 $$ F_{m} = \frac{V\chi }{{\mu_{o} }}\left( {\vec{B} \cdot \nabla } \right)\vec{B}, $$

5 $$ F_{d} = \frac{2\pi \eta L\upsilon }{{\left[ {\ln \left( {\frac{L}{{2\sqrt {S/\pi } }}} \right) + \ln 2 - 0.5} \right]}}, $$

6 $$ F_{f} = \mu_{s} W = \mu_{s} gnm, $$

where *V* is the total volume of particles in the particle chain; $\chi$ is the susceptibility of magnetic particles; *μ*_*o*_ is the vacuum permeability; *η* is the dynamic fluid viscosity; $\upsilon$ is the tumbling velocity; *S* is the cross-section of magnetic chains; *n* is the number of particles in the chain; *m* is the mass of a single magnetic nanoparticle; and *μ*_*s*_ is the wet friction coefficient, which is proportional to the tumbling velocity after balancing the torque required to shear the intervening fluid layer, i.e., magnetic torque, with the friction torque, as follows^39^:

7 $$ \mu_{s} = \frac{\eta }{hP}k\overset{\lower0.5em\hbox{$\smash{\scriptscriptstyle\frown}$}}{\upsilon } , $$

where *h* is the gap between the chains and working plane; *P* is the pressure on the magnetic particle during tumbling; $$\overset{\lower0.5em\hbox{$\smash{\scriptscriptstyle\frown}$}}{\upsilon }$$ is the fluid velocity between the chains and a nearby surface that is equal to circumferential velocity, $$\upsilon_{\omega }$$; and *k ≡ η/hP*. Therefore, the friction force can be recalculated as follows.

8 $$ F_{f} = k\upsilon_{\omega } W = k\omega RW $$

Finally, the tumbling velocity can be derived using magnetic, friction, and drag force balancing.

9 $$ \upsilon = \frac{{k\omega RW + \frac{V\chi }{{\mu_{o} }}\left( {\vec{B} \cdot \nabla } \right)\vec{B}}}{2\pi \eta L}\left[ {\ln \left( {\frac{L}{{2\sqrt {S/\pi } }}} \right) + \ln 2 - 0.5} \right] $$

### Experimental setup

Nine power suppliers are used to control the coil currents, including five MX15 units and four 3001iX units from AMETEK, USA. The cooling system consisting of a chiller (Lauda VC7000, Germany) with copper pipes surrounding each coil is used to ensure that the coil temperature does not exceed 40 °C. The details of the coil temperature as a function of the applied current are shown in Fig. S3. In which, the temperature change rates with respect to applying voltage are 1.5 °C/min, 4 °C/min, and 9.7 °C/min at 10 V, 20 V, and 30 V (as shown in Fig. S3a), respectively. The resistance change rates with respect to temperature are 0.0075Ω/ ^o^C as shown in Fig. S3b. In this study, a digital single-lens reflex (DSLR) camera (Canon EOS 600D) with a macro-lens (Canon EF 100 mm f/2.8) was used to observe the particles. Hamamatsu biplane microfocus X-ray sources (90 kV; focal spot size of 7 µm) was used to verify the visibility of MNPs under X-ray monitoring by considering further in vivo experiment. The whole system is controlled via LabVIEW from an Intel Core i7 3.4 GHz computer.

## Results and discussion

### Disaggregation of nanoparticle chains

To accomplish targeted drug delivery, the nanorobots must not only be capable of gathering and moving together but also have the capacity to break up into the original particles, allowing them to enter microvascular vessels or penetrate a tumor. Therefore, the disaggregation of particle chains or a swarm of nanoparticles by repulsing a nearby surface or wall is necessary to achieve this task. The resultant clustered nanoparticles by the magnetic fields are shown in Fig. 4, c, and d. By applying an alternative uniform magnetic field and a gradient field perpendicular to the working plane, a repulsive force is induced among the particle chains, consequently propelling them away from each other, as shown in Fig. 4e. The magnetic interaction force, $F_{i}^{m}$, exerted on the *i*th particle chain in the midst of *N − 1* chains can be described as follows^40^:

10 $$ F_{i}^{m} = \frac{{3\mu_{o} }}{4\pi }\sum\limits_{j = 1,i \ne j}^{N} {\left[ {\frac{{m_{i} m_{j} }}{{r_{ij}^{4} }}\left\{ {\left( {1 - 5\left( {\hat{m} \cdot \hat{r}_{ij} } \right)^{2} } \right)\hat{r}_{ij} + 2\left( {\hat{m} \cdot \hat{r}_{ij} } \right)\hat{m}} \right\}} \right]} $$

where $\hat{m}$ is the unit vector of the magnetic field; *m*_*i*_ and *m*_*j*_ are the induced magnetic moments of the *i*th and *j*th chains, respectively; and $\hat{r}_{ij}$ is the unit vector between the centers of the two aforementioned chains. The gradient field is applied to confine the chains in the working plane, and together with the alternative uniform magnetic field, the chains are separated into particles by the dipole–dipole repulsive force. The magnetic force acting on the *i*th chain can be calculated by Eq. (4). Figure 4f demonstrates the successful disaggregation of the proposed swarm structure utilizing the foregoing technique.

### Velocity of nanoparticles

Velocity is an important metric for evaluating the efficiency of locomotion. Here, the most recent locomotion techniques, including conventional tumbling motion, gradient pulling motion, vortex-like swarm motion, and enhanced tumbling motion, are implemented and compared to evaluate any improvements in the proposed method.

A nanocluster concentration-based tracking algorithm is applied to measure the velocity of nanoparticle clusters^28^. First, the intensities of specific particle concentrations are investigated for tracking the centroids of clusters with different concentrations, i.e., 8000, 4000, 2000, 1000, 500, 250 μg/mL, and 0 μg/mL (Fig. S4a). The other parameters (e.g., ambient light, focal length, and contrast) are fixed to maintain the same conditions during the measurement. The velocity test was performed in a polydimethylsiloxane (PDMS) channel with dimensions 20 × 5 × 5 mm^3^, as shown in Fig. S4b. The red dot and varying yellow color intensities represent the centroid and nanocluster concentration after tracking and reconstruction, respectively. In this experiment, a drop of nanoparticle suspension (20 μL) with a concentration of 6.67 mg/mL is introduced into the channel filled with the PBS solution. First, the dispersed particles are gathered by the EMA system before locomotion.

**Figure 5:**
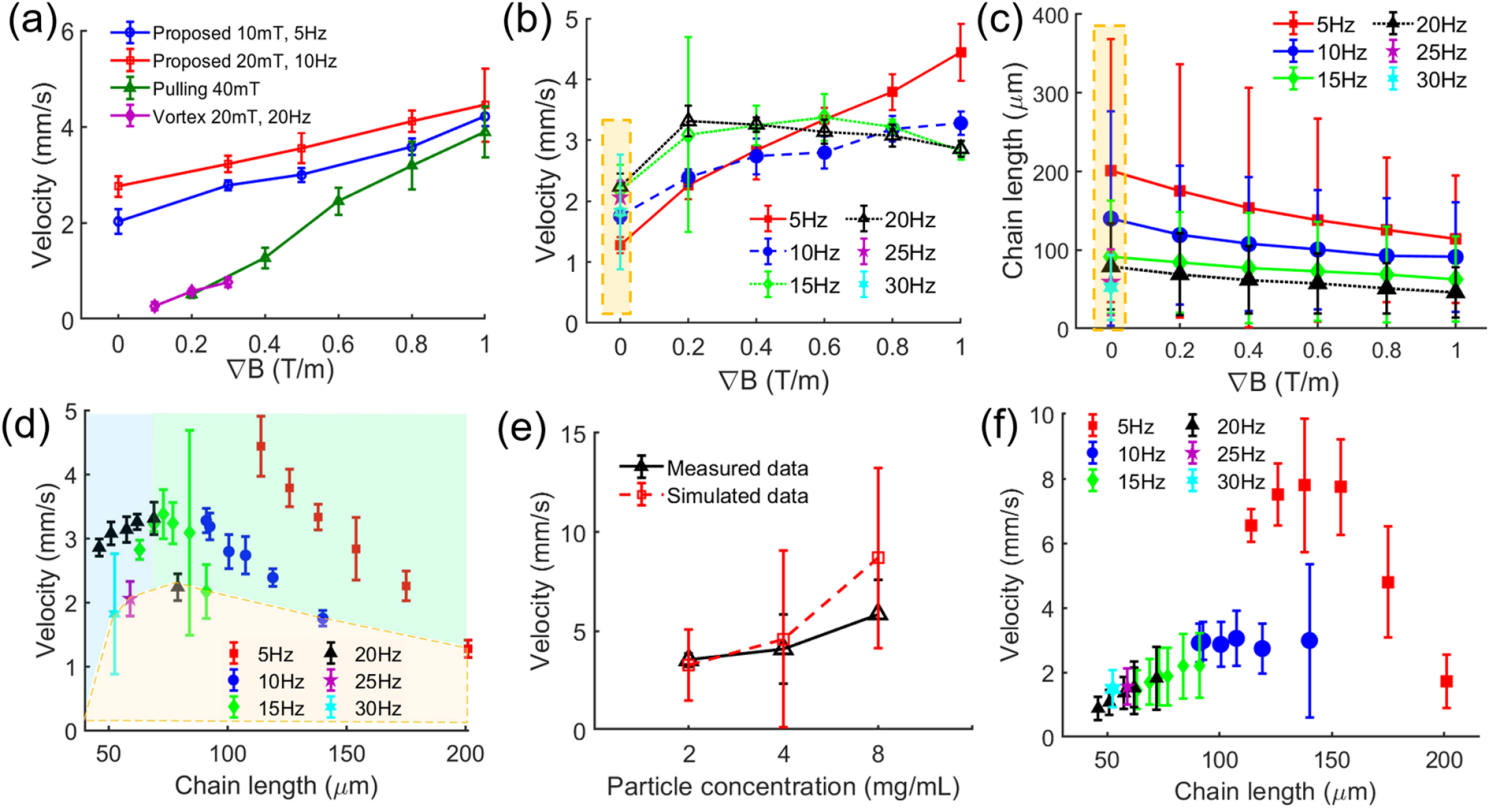
Locomotion test of nanoparticles. (**a**) Experimental results of different particle control methods including conventional tumbling motion, gradient pulling motion, vortex-like swarming motion, and proposed enhanced tumbling motion. (**b**) Measured moving speed of enhanced tumbling motion at constant 10 mT field with various rotating frequencies (5, 10, 15, 20, 25, and 30 Hz) and gradient field strengths (0, 0.2, 0.4, 0.6, 0.8, and 1 T/m). **c** Estimated length of chains under conditions similar to (**b**). Orange bounded regions in (**b**,**c**) are data for conventional tumbling motion with zero gradient field. (**d**) Plot of moving velocity with respect to chain length; orange region represents data for conventional tumbling motion, green is gradient-active region, and blue is inactive region for both rotating frequency and gradient. (**e**) Measured and simulated data of moving speed of chain-like cluster with different particle concentrations. (**f**) Simulation results of moving speed dependent on rotating frequency and gradient under conditions similar to (**b**,**c**,**d**).

The velocities of clusters with conventional tumbling, pulling, and enhanced tumbling motion and vortex-like swarming using the gradient field (more details on the vortex-like swarming motion used in this work are found in the Supplementary material) are shown in Fig. 5a. For each method, the experiments are repeated at least three times.

First, the conventional tumbling motion is implemented. Two conditions of the magnetic field and rotating frequency are used: 10 mT, 5 Hz, and 20 mT, 10 Hz, respectively. At 20 mT and 10 Hz (first point on the red line in Fig. 5a), the conventional tumbling motion (i.e., zero gradient) exhibits a moving velocity of 2.76 ± 0.21 mm/s. At 10 mT and 5 Hz (the first point on the blue line), the clusters exhibit a slightly lower velocity of 2 ± 0.26 mm/s. In the enhanced tumbling motion, the magnetic field gradient with different strengths (0.3, 0.5, 0.8, and 1 T/m) has been applied to move the clusters with similar field strength and frequency applied to the conventional tumbling motion. The enhanced tumbling motion shows a virtually linear proportion to the change in the field gradient. At 20 mT and 10 Hz (red line), the moving velocity of the clusters linearly increases from 3.2 ± 0.17 mm/s at 0.3 T/m to 4.5 ± 0.8 mm/s at 1 T/m; at 10 mT and 5 Hz (blue line), the velocity is slightly lower, i.e., from 2.8 ± 0.1 mm/s at 0.3 T/m to 4.2 ± 0.2 mm/s at 1 T/m. The results of the gradient pulling of magnetic clusters are demonstrated by the green line (Fig. 5a). The moving velocity of the magnetic clusters by gradient pulling motion accelerates faster than that of the enhanced tumbling motion. This is evident because only the magnetic pulling force exerted on the cluster causes this motion; hence, the significant change in locomotion speed is in response to the field gradient variation. Gradient pulling is less efficient than the enhanced tumbling motion with a maximum speed of 3.8 ± 0.53 mm/s at an applied field of 40 mT and a gradient of 1 T/m. In the conventional tumbling motion of particle chains, frequency performs a vital function in the moving velocity.

In the enhanced tumbling motion, as indicated by the gap differences between the red, blue, and green lines (i.e., the contribution of rotating frequency) and the differences among the lines (i.e., the contribution of the field gradient), the contribution of the changing field gradient to the increase in the moving velocity is higher than that of the rotating frequency.

The results of the moving velocity caused by the vortex-like swarm are represented by the purple line in Fig. 5a. The locomotion mechanism of the vortex-like swarm is described in “Materials and methods” section in supplementary material and Fig. S5. In this measurement, the vortex of particles was first generated by applying an in-plane rotating field with an amplitude of 20 mT and frequency of 20 Hz. The gradient fields, which vary from 0.1 to 0.3 T/m, are then applied to the vortex for locomotion. As mentioned in the Supplementary Material, the higher field gradient breaks the vortex structure. The moving velocity of the vortex-like structure is proportional to the gradient field and can achieve a maximum speed of 0.78 ± 0.1 mm/s at 0.3 T/m. These results demonstrate the improvement in the enhanced tumbling locomotion efficiency, which is approximately 20% higher than that of the gradient pulling motion using both magnetic and gradient fields. It is also virtually twice the locomotion speed of conventional tumbling motion at 1 T/m, and more than five times faster than the vortex-swarming motion.

### Effects of control parameters

#### Magnetic field effects

To thoroughly investigate the effect of gradient and rotating frequency on cluster motion in the proposed method, further experiments on the magnetic cluster locomotion are implemented by fixing the intensity of the magnetic field (10 mT) and varying the applied field gradient (0, 0.2, 0.4, 0.6, 0.8, and 1 T/m) and rotating frequency (5, 10, 15, 20, 25, and 30 Hz). The measured moving velocities of the magnetic clusters under each condition are shown in Fig. 5b. Here, the linear increment of speed with the frequency and gradient changes similar to the red and blue lines in Fig. 5a is no longer observable. First, consider the zero-gradient point (i.e., the conventional tumbling motion). The chain-like clusters show a gradual increase in the locomotion speed from 1.3 ± 0.14 mm/s at 5 Hz to a maximum of 2.2 ± 0.2 mm/s at 20 Hz where the step-out frequency occurs. After the step-out frequency, any further increase in frequency results in a decrease in locomotion speed down to 2 ± 0.27 mm/s and 1.8 ± 0.94 mm/s at 25 and 30 Hz, respectively. When the gradient field is applied (i.e., the enhanced tumbling motion), the moving velocity of the magnetic clusters can only maintain a linear proportionality to the gradient at 5 Hz (red solid line in Fig. 5b) with a maximum of speed of 4.4 ± 0.47 mm/s at 1 T/m. At higher frequencies, this relationship becomes non-linear at 10 Hz with a maximum speed of 3.3 ± 0.2 mm/s at 1 T/m (blue dashed line in Fig. 5b); it even peaks out with speeds of 3.4 ± 0.4 mm/s at 15 Hz and 0.6 T/m (green dotted-dashed line) and 3.3 ± 0.25 mm/s at 20 Hz and 0.2 T/m (black dotted line).

To explain this phenomenon, recall the interaction forces among the particles in the chain-like cluster in Eq. (10). In the experiments, the magnitudes of the magnetic dipole moments of particles are unchanged; hence, the dipole–dipole interaction forces are constant. Meanwhile, the rotating frequency increment increases the centrifugal force exerted on the rotating chains, especially on the outermost particles of the chain where the magnetic bond is relatively weak. This continues until the centrifugal force becomes sufficient to break the dipole–dipole interactions and reduce the magnetic chain size. Consequently, the magnetic force and torque acting on the chains also decrease, resulting in a lower moving speed. In addition, the magnetic force induced by the gradient field exerted on each particle not only steers the particles to move to the desired direction faster but also contributes to the breakage of particle interaction, particularly among the outermost particles (Fig. S6).

#### Particle size affects

It is exigent to derive an exact mathematical model of the creation of the chain-like clusters in the enhanced tumbling motion with respect to the rotating frequency and magnitude of the applied magnetic field and field gradient. Hence, the authors decided to experimentally investigate the magnetic chain size from the recorded videos using an image processing technique (as shown in Fig. S7a). The mean values of the chain length and diameter were calculated from at least 300 chains under each condition to ensure meaningful results. The estimated magnetic chain lengths and diameters under similar experimental conditions in Fig. 5b are demonstrated in Fig. 5c and Fig. S8, respectively. It is apparent that the length and diameter are inversely proportional to the rotating frequency and gradient. For instance, the chain lengths gradually reduce from 201 ± 167 to 52.5 ± 41.5 µm at 0 T/m and 5–30 Hz as shown by the orange bounded region in Fig. 5c. When a range of 0.2–1 T/m gradient is applied, the chain lengths also decrease from 175 ± 161, 119 ± 88, 77 ± 64, and 62 ± 52 µm to 114 ± 81, 91 ± 70, 63 ± 54, and 46 ± 32 µm at 5, 10, 15, and 20 Hz, respectively. Based on these, if the relationship of the chain size relative to the moving speed (Fig. 5d) is considered, the conventional tumbling motion reaches its peak when the chain length is 79 ± 55 µm. This indicates the active region of rotating frequency where the moving capacity of the magnetic clusters continues to well conform with the frequency increment. When the chain length exceeds 114 ± 81 µm, the field gradient contribution is significant if the moving velocity and gradient at 5 Hz (0–1 T/m) are linearly dependent and continue to follow the trend at 10 Hz (0–0.2 T/m), as shown in Fig. 5b,d. When the chain length is 70–110 µm, the gradient continues to contribute to the accretion of locomotion efficacy despite the chain length reduction. The foregoing causes the clusters to have a lower efficiency even when the applied gradient and rotating frequency are high compared with the clusters with a suitable size under a small gradient and frequency. For instance, the moving speeds at a gradient of 0.8 T/m and rotating frequencies from 10 to 20 Hz (3.2 ± 0.2, 3.2 ± 0.1, and 3.1 ± 0.18 mm/s) are smaller than those of 5 Hz and 0.6 T/m (3.3 ± 0.2 mm/s).

#### Concentration affects

The moving speed of the clusters is experimentally found to rely not only on the magnetic and gradient field strengths and rotating frequency but also on particle concentration. In this experiment, a nanoparticle suspension (30 μL) with various particle concentrations (2, 4, and 8 mg/mL) is added into the aforementioned PDMS channel filled with a PBS solution. The proposed method is then applied to control the locomotion of particles with a magnetic field of 30 mT, a frequency of 5 Hz, and a gradient field of 1 T/m. The velocities are shown in Fig. 5e and Fig. S8, in the Supporting information. The moving speed of the clusters increases as the particle concentration increases (2, 4, and 8 mg/mL). In Fig. S7, b–c, the length and diameter of the chains are also analyzed from the recorded videos as shown in Fig. S7a. The increments in both length and diameter of the magnetic chains in a similar global field demonstrate that the increase in particle concentration improves the probability of the aggregation of higher volumes of chain-like clusters that can lead to higher magnitudes of magnetic torque and force exerted on the chains. This result can be used as a reference for selecting a sufficient injection concentration of nanoparticles for in vivo experiments to maintain high controllability and access rate. Interestingly, the two tumbling motions under 10mT, 5 Hz in Fig. 5a,b have different moving speeds as 2 ± 0.26 mm/s and 1.3 ± 0.14 mm/s, respectively. The difference in the two experiments is the result of different particle concentrations caused by the dispersion property of the particles and device accuracy.

### Simulation model validation

To derive and verify the MNP enhanced tumbling motion model for further analysis and control design, the numerical simulations of magnetic nanoparticles with respect to the above experimental conditions were performed based on the aforementioned mathematical model. Here, the chain-like cluster is assumed as a rod with a length and diameter corresponding to those of the chains. Figure 5f shows the simulation results of the moving test under the following conditions. The magnetic field is 10 mT, the field gradient values are 0, 0.2, 0.4, 0.6, 0.8, 1 T/m; and the rotating frequency values are 5, 10, 15, 20, 25, and 30 Hz. In general, the simulation results well agree with the logical trend of the experimental results mentioned above including the increment of moving speed with the increases in frequency (i.e., 1.7–2.986 mm/s at a frequency of 5–10 Hz) and gradient (1.7–7.8 mm/s at a gradient of 0–0.6 T/m). However, the proposed model is extremely sensitive to the chain size (i.e., length and diameter) than the gradient because the simulated velocity can rapidly reach the peak when the chain length and diameter are less than 140 and 67.5 µm, respectively. The simulation results of moving velocities of chains with different particle concentrations (red dashed line in Fig. 5e) well conforms with the experimental data although the simulated speeds exceed 3.3 ± 1.8, 4.6 ± 4.45, and 8.7 ± 4.6 mm/s at 2, 4, and 8 mg/mL, respectively.

### Targeting performances in a channel

**Figure 6:**
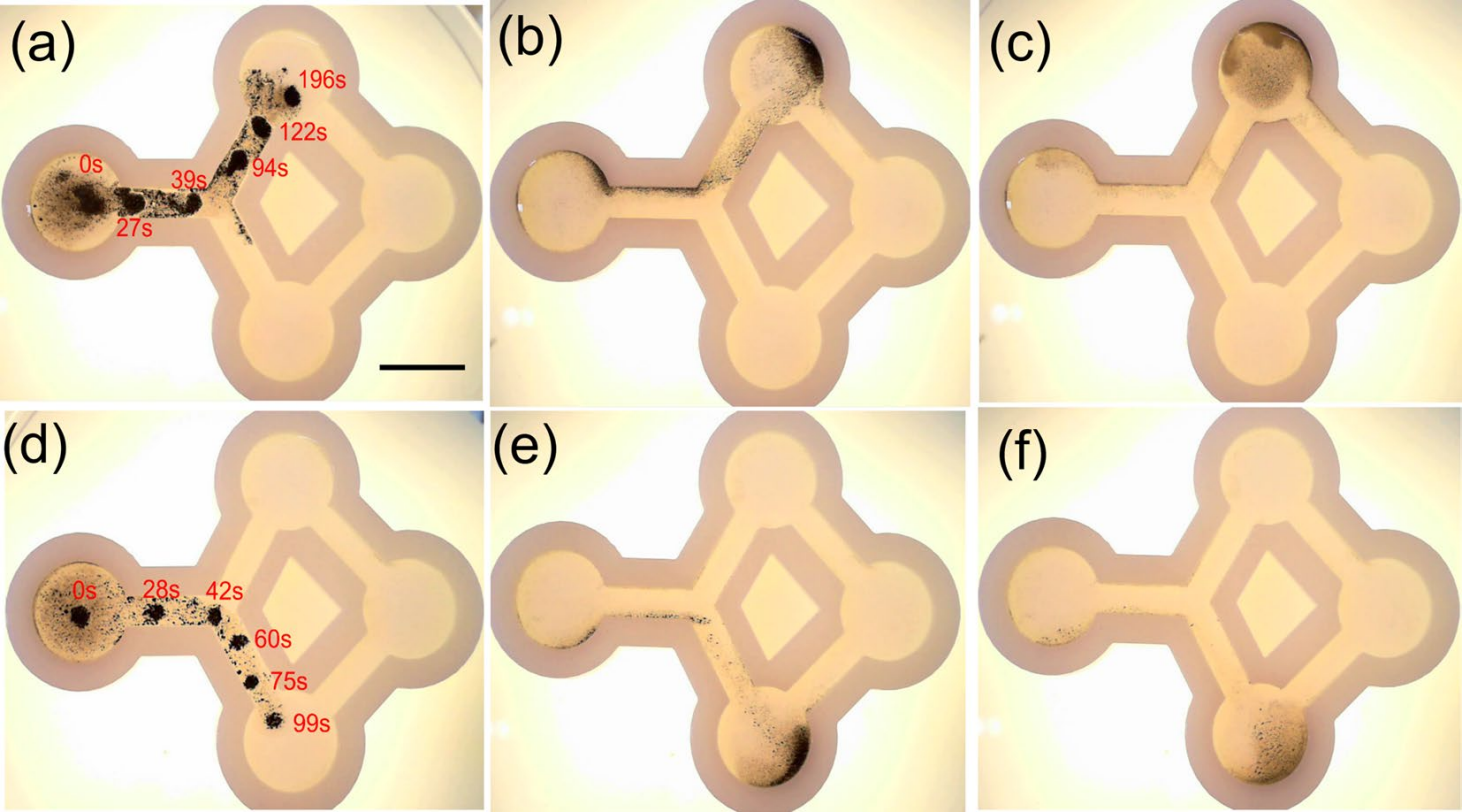
Time-lapse image of targeting tests in channel using vortex-like swarm toward top (**a**) and bottom (**d**) target sites. The red numbers at each point indicate the time when vortex is located; (**b**,**e**) enhanced tumbling motion toward top and bottom target sites, respectively. (**c**,**f**) Disaggregation of pattern after targeting top and bottom of channel. Scale bar: 10 mm.

Two approaches to particle manipulation are used to demonstrate targeting efficiency through the two-branch channel (Fig. 6). The desired cluster paths start from the initial location (circular shape on the left of the figure) through the upper/lower branch to the target site (circular shape located at the top/bottom) with a total travel distance of 44 mm. In this experiment, a 10 μL droplet of nanoparticle suspension is used. Figure 6a,d, demonstrates the time-lapse targeting process using the vortex-like swarm motion at the upper and lower sides, respectively. The magnetic field, gradient, and rotating frequency used are 20 mT, 200 mT/m, and 20 Hz, respectively. In this experiment, the wall effect is investigated by approaching the channel wall during cluster locomotion. When the cluster approaches close to the surface (at t = 27 s in Fig. 6a,d), some fragments tend to attach to the wall and are remain there as the drag coefficient increase near the wall, resulting in an imbalance of the fluidic interaction forces acting on the structure. The pattern extended to a critical length before splitting into smaller vortices (t = 40 s; supplemental video). However, these small vortices were partially reunited by stop-and-go locomotion (t = 94 s in Fig. 6a). The structure then reached the upper and lower target sites after 196 and 99 s, respectively. The slightly modified tracking algorithm was used in this case to measure the access rate of particles to the target as a function of their concentration. The access rate when targeted the upper area was 85%, while that of the lower target was only 70%; the remaining particles were left inside the channel. Figure 6b,e illustrate the targeting process taking advantage of the enhanced tumbling motion towards the upper and lower targets. The elongation of the swarm in this locomotion method is significant; the head reaches the target site while the tail remains at the initial site. However, it exhibits an extremely fast target time compared with the vortex-like swarm locomotion using a gradient. In the latter, the target times were 44 s for upper site targeting and approximately 1 min for lower site targeting due to its high moving velocity. The targeting rates of tumbling motion using a two-branch channel are 95.5% for upper site targeting and 92.8% for lower targeting, respectively. Disaggregation was implemented after targeting the top and bottom sites, as shown in Fig. 6c,f.

**Figure 7:**
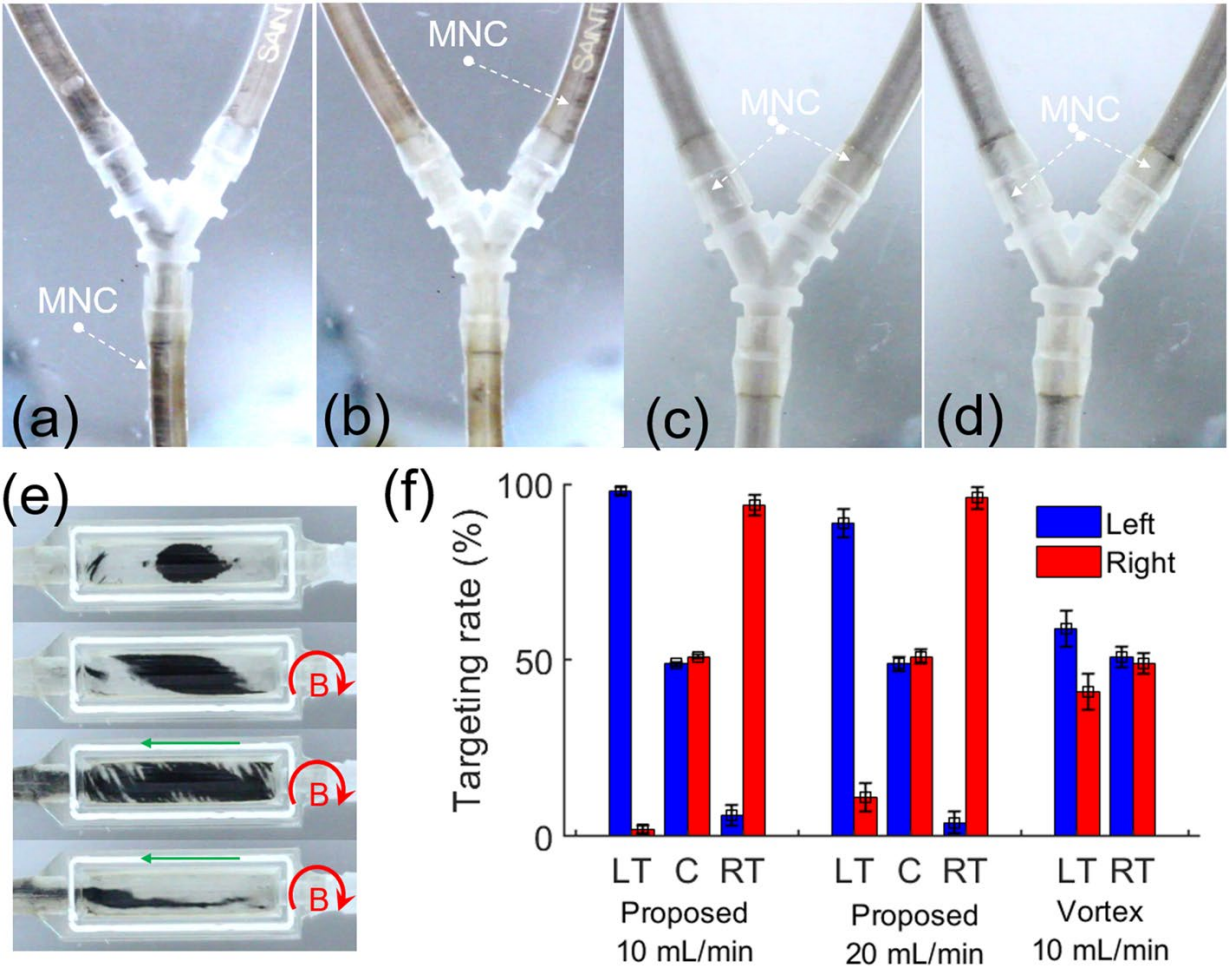
Targeting performance of MNR in a channel with flow condition. A frame capture of targeting test using the proposed method to (**a**) left channel, (**b**) right channel under a flow rate of 20 mL/min. A frame capture of targeting test using vortex-like swarm to (**c**) right and (**d**) left channel under a flow rate of 10 mL/min. (**e**) A time-lapse vortex formation test under a flow rate of 10 mL/min, where the green arrow represents the flow direction. (**d**) Recorded targeting rate of the MNR using the proposed method under a different flow rate of 10 mL/min and 20 mL/min and vortex-like swarm with a flow rate of 10 mL/min. Where LT, C, and RT are left targeting, control, and right targeting, respectively. (Please refer to the supplementary videos for more details of these results).

The dynamic performance of the proposed control method under the flow condition was evaluated in comparison with a vortex-like swarm method. A fluidic channel mimicking the human’s portal vein having one inlet and two outlets consisting of silicone tube (inner diameter of 2.38 mm, Tygon, USA) and a plastic Y-tube connector (PPY02, Azlon®) is fabricated (as shown in Fig. S9). The flow rate is controlled by the multichannel syringe pump (Fusion 4000, Chemyx, USA). To investigate the effects of the flow on the locomotion of the MNR, the targeting performances are considered with different flow rates of 10 mL/min and 20 mL/min. The tumbling motion was applied in the targeting test with 30 mT, 1 T/m, and a rotating frequency of 10 Hz. Three targeting experiments to the left (i.e. targeted to the left channel with the applied rotating field on the XZ plane, counterclockwise and θF:180o, αF: 0o), to the right (i.e. targeted to the right channel with the applied rotating field on the XZ plane, clockwise and θF:0o, αF: 0o), and the control (no magnetic field applied) were conducted. The results show that the targeting of the MNR is well performed against the flow with a targeting rate of over 89% (as shown in Fig. 7). It is obvious if we compare it to the experimental results of no field conditions (as shown in Fig. 7f), where the targeting rates to the left and right are 49% and 51%, respectively. At a flow rate of 10 mL/min, the proposed method demonstrated good targeting results to the left and right sides, with rates of 98% ± 1% and 94% ± 3%, respectively. At a faster flow rate of 20 mL/min, the targeting performance (as shown in Fig. 7a,b) slightly decreases with access rate to the left and right channels of 89% ± 4% and 96% ± 3%, respectively. Figures 7c,d show the targeting of the vortex-like swarm under a flow rate of 10 mL/min. The magnetic field applied in this test is similar to the previous experiment in a static environment (Fig. 6a,d) with 20mT, 200mT/min and 20 Hz. The targeting performance of vortex-like swarm is relatively poor with both left and right sides, with a targeting rate of 59% ± 5% and 49% ± 3%, respectively. Moreover, to evaluate the formation of vortex-like swarm under the flow condition, another experiment was carried out in “I” channel size of 5 mm in width, 20 mm in length, and 5 mm in height, which has one inlet and one outlet as shown in Fig. 7e. A droplet of MNR solution is injected into the channel (first top image in Fig. 7e), following by the applying of the magnetic field to form the vortex. Here, the vortex formation in a narrow area shows another disadvantage of this swarming in practice where the MNR is contacted with the vessel wall results in a shearing torque that tends to elongate the swarm (second top image in Fig. 7e). As shown in Fig. 7e (two bottom images), when the flow is on, it is clear that the vortex cannot be formed and MNRs are easily flush away (please refer to the supplementary video for more details). Although the experimental results under the flow condition can validate the better performance than the conventional vortex-like swarm motion, further validation with higher blood flow conditions needs to be conquered under sophisticated setups.

## Conclusions

In this paper, we have presented the theory, simulation, and experimental manipulation of nanoclusters using the enhanced tumbling motion with a gradient magnetic field. The experimental results of the proposed manipulation approach were compared with other methods and verified that the proposed method can perform high moving velocities and high controllability. The contributions of the gradient field strength, the frequency of the rotating field, and MNP cluster size to the moving velocity are demonstrated to be quantitatively comparable; hence, the performance of the enhanced locomotion can be explained in the analytical backgrounds. In the formulated model, the magnetic clusters are modeled as magnetic rods, and the hydrodynamic interactions are only partially considered (only Stokes’ drag acting at the interface between the fluid and magnetic clusters are taken into account). The simulation results agree well with the measurement results. However, the model shows a relatively strong dependence on the chain size rather than the gradient strength of the applied field. The swarm structures traversing the channel with the proposed manipulation method with a high access rate demonstrate the potential of the proposed method for the application of targeted drug delivery in practice. In particular, the enhanced tumbling motion could be of great benefit in vivo experiments in terms of moving velocity and access rate. However, the access rate of this locomotion method needs to be verified by in vivo experiments as the next step to fully validate this method. Particle visualization is also necessary to obtain high performance of the targeting task as well as navigation of nanoparticles in combination with anticancer drugs.

## Supplementary Information

Supplementary Information 1.

Supplementary Video 1.
