# Supplementary material for: Locomotion and disaggregation control of paramagnetic nanoclusters using wireless electromagnetic fields for enhanced targeted drug delivery
Source: Sci Rep. 2021 Jul 23;11:15122. doi: 10.1038/s41598-021-94446-4 (PMC8302636; doi:10.1038/s41598-021-94446-4)
Supplement: Supplementary file 1 — Supplementary Information 1. [file 41598_2021_94446_MOESM1_ESM.docx]

Supplementary information

**Section S1. Control principle and optimal coil placement in EMA System**

The EMA system for nanorobots generates magnetic fields and gradients with the desired direction and intensity within the workspace through the superimposition of the magnetic field from multiple coils ^1^. The current ($\boldsymbol{i}$) applied to the multiple electromagnetic coils of the EMA system is calculated via a pseudo-inverse based on the desired magnetic field ($\mathbf{B}$) and magnetic force ($\mathbf{F}$**)**. It can be expressed as follows.

$\boldsymbol{i}=\mathcal{A}_{B, F}{(M, P)}^{\dagger}\left[ \begin{matrix} \mathbf{B} \\ \mathbf{F} \end{matrix} \right]=\left[ \begin{aligned} \begin{matrix} \mathbf{B}(P) \\ \mathbf{M}^{T}\frac{\partial\mathbf{B}(P)}{\partial x} \end{matrix} \\ \mathbf{M}^{T}\frac{\partial\mathbf{B}(P)}{\partial y} \\ \mathbf{M}^{T}\frac{\partial\mathbf{B}(P)}{\partial z} \end{aligned} \right]^{\dagger}\left[ \begin{matrix} \mathbf{B} \\ \mathbf{F} \end{matrix} \right]$ (S1)

The 3×n matrices of $\mathbf{B}(P)$, i.e., $\frac{\partial\mathbf{B}(P)}{\partial x}$, $\frac{\partial\mathbf{B}(P)}{\partial y}$, and $\frac{\partial\mathbf{B}(P)}{\partial y}$, are magnetic fields and gradient matrices generated in n coils per unit current at point ($P$) of the workspace; $\mathcal{A}_{B, F}(M,P)\in\mathbb{R}^{6\times n}$ is the actuation matrix of the EMA system. The pseudo-inverse of $\mathcal{A}_{B, F}(M,P)$ uses the singular value decomposition, $\mathcal{A=}\mathbf{U}\sum\mathbf{V}^{T}$, where $\mathbf{U}$ is the n × n orthonormal matrix whose columns are the singular vectors of $\mathcal{A}\mathcal{A}^{T}$; $\mathbf{V}$ is the n × n orthonormal matrix whose columns are the singular vectors of $\mathcal{A}^{T}\mathcal{A}$, and $\sum$ is the 6 × n diagonal matrix whose diagonal entries, $\sigma_{i}$, are singular values of $\mathcal{A}$. The magnetic objects are aligned according to the magnetic fields generated by the EMA system. Therefore, if the magnetic fields do not abruptly change, the magnetization ($\mathbf{M}$) in equation (S3) coincides with the magnetic fields ($\mathbf{B}$).

The design of the EMA system consists of several requirements and conditions:

- Stationary electromagnetic coils were applied for safety in clinical applications. In addition, a soft magnetic core was inserted into the coil to generate stronger magnetic fields than the air core. In the coil configuration, the axis of coils passes through the center of the workspace, and all coils are equidistant from the center of the workspace. The workspace of the EMA system resembles a sphere. The workspace size, the distance between the center of the workspace and coil, and the coil parameter were determined to fit the target object (Table S1).
- Magnetic targeting in three-dimensional (3D) space is necessary for nanorobots to gain access into a blood vessel with a complicated 3D path. Nanorobots move along the blood vessels; hence, for 3D targeting, the EMA system must have motions with five (two rotational and three translational) DOFs. Based on the condition of the coil configuration under design condition (1) of the EMA system, at least eight are required to satisfy the five DOF motions ^2^.
- The region of coil placement was designed to allow the access to the target object, operators, and two x-ray imaging devices. As shown in Figure S1 and listed in Table S2, the region is divided into upper and lower regions. The upper region was set up to allow access to the target object, imaging devices, and nanorobot injection tools. Thus, at the upper region, the coil can be arranged along the x-axis with an angle range of 0°–10° from the xy plane and 45°, 135°, 225°, and 315° around the z-axis. The lower region has a range where the coil does not interfere geometrically; its angle range is −22.5°–0° from the xy plane and 0°– 360° around the z-axis. In this coil placement region, up to nine coils (n = 9) can be placed without any interference among them. In this study, we selected nine coils as the number of coils in the EMA system to enhance the maximum generated gradient field and utilize redundancy to avoid the waste of power ^3^.

To satisfy the above conditions, we attempted to obtain an optimal coil configuration suitable for the 3D targeting of nanorobots through an optimization routine. This routine is performed using parameters, such as the number of coils, region of coil placement, interference among the coils, and the actuation matrix from equation (S3). In particular, the singular value of the actuation matrix was a vital factor in predicting the performance of the EMA system ^4,5^. For example, $\sigma_{6}/\sigma_{1}$(a condition number) represents the isotropy in terms of both the magnetic field and force in the workspace for the EMA system, and the EMA system provides more isotropic control within the workspace as the condition number approaches one. Moreover, the minimum singular value ($\sigma_{6}$) in the actuation matrix can be used as an indicator of the workspace strength of the EMA system. In the comparison of different coil configurations, the high minimum singular value in the least efficient direction maximizes the strength of the workspace in the EMA system. As another factor for confirming the performance of the EMA system, we used the determinant ($\prod_{i=1}^{6} \sigma_{i}$) of the actuation matrix calculated from singular values to obtain the optimal coil configuration. The authors are of the opinion that the determinant of the actuation matrix not only includes all singular values of the actuation matrix but also directly involves the inverse matrix in the coil current calculation given by equation (S3). Therefore, this determinant is an index closely related to the performance of the EMA system. For instance, a system with a specific coil configuration with a higher determinant index can result in better performance in terms of the force and field. The optimization of coil placement is sequentially performed through the creation of coil configurations, generation of unit current magnetic fields and gradient matrices, calculation of the determinant for the actuation matrix, and comparison of determinants from among the candidates for the coil configuration. Each step is described as follows.

**Step (1)** Candidates for coil configuration are formed based on the coil placement region. Here, the coil arrangement causing the interference among the coils is excluded.

**Step (2)** In each possible coil configuration, $\mathbf{B}(P)$, $\frac{\partial\mathbf{B}(P)}{\partial x}$, $\frac{\partial\mathbf{B}(P)}{\partial y}$, and $\frac{\partial\mathbf{B}(P)}{\partial y}$, which are submatrices of the actuation matrix ($\mathcal{A}$), are obtained through numerical simulation. The magnetic fields generated by the coil with a soft magnetic core required a non-linear simulation; hence, we proceeded with the optimization routine using COMSOL (COMSOL Inc., MA, USA) and MATLAB (MathWorks, MA, USA).

**Step (3)** For a given coil configuration, the determinant ($\prod_{i=1}^{6} \sigma_{i}$) of the actuation matrix ($\mathcal{A}$) was analyzed at 27 positions in a 20 mm spherical workspace and 26 orientations of magnetization ($\mathbf{M}$) at each point. As a representative factor of the given coil configuration, the smallest determinant (i.e., the worst case) was obtained at 27 positions in the workspace of the given coil configuration.

**Step (4)** Steps (1)–(3) were repeated for all candidates, and the minimum determinant values in the workspace for each coil configuration were compared.

Among the coil configurations listed in the order of decreasing minimum determinant values in the workspace through the above optimization routine, the optimized coil configurations are described in Figure S2 and listed in Table S3. The EMA in this study is designed by considering the animal experiments under X-ray, it has distinguished with the MiniMag^6^ in terms of number of coils (eight vs nine), workspace (30mm × 30mm vs. 120mm × 120mm), maximum magnetic field (50mT with 5T/m vs. 174mT with 5T/m), and eventually X-ray device compatibility.

**Section S2.** **Swarm motion vortex-like structure utilizing gradient field**

The motion of small particles in vortex-induced flow that has been studied by Lecouna et al. is broadly used ^7^. The particle's velocity in the vortex can be described as follows:

$v=u+St\left( \frac{3}{2}\varepsilon-1 \right)\left( u\cdot\nabla u-g \right)+O\left( St^{\frac{3}{2}} \right)$ (S2)

$\begin{aligned} v_{r}=St\left( \frac{3}{2}\varepsilon-1 \right)\left( -\frac{u_{\theta}^{2}\left( r \right)}{r}-g_{r}\left( \theta\right) \right)+O\left( St^{\frac{3}{2}} \right) \\ v_{\theta}=u_{\theta}\left( r \right)-g_{\theta}\left( \theta\right)St\left( \frac{3}{2}\varepsilon-1 \right)+O\left( St^{\frac{3}{2}} \right) \end{aligned}$ (S3)

where v is the particle velocity; *v_r_* and *v_θ_* are the tangential and radial velocities of the particle, respectively; *St* is Stokes number; *g_r_* and *g_θ_* are the tangential and radial components of the gravitational acceleration vector (g), respectively; *ε* is the density ratio; and *u* is the flow velocity of the particle inside the vortex core (r < R) in which the vortex is assumed to undergo rigid body rotation ^8^. Therefore, *u_θ_* is linearly distributed along the radial direction and can be expressed as

$u_{\theta}=\frac{\Gamma_{o}r}{2\pi R^{2}}$ (S4)

where *Г_o_* is the original circulation of the vortex. After the vortex-like structure has been steadily generated, as shown in Figure S4(a), the gradient field is then applied to efficiently and accurately control the translation motion of the vortex with a strong magnetic force (Figure S4 (b)). This is because the volume of the total number of particles in the vortex core remarkably increases according to (4). Although the gradient field can efficiently drive the vortex, it can affect the vortex structure due to the unbalanced resultant of the magnetic force induced by the strong gradient, vortex inward force, and drag force on the particles around the vortex. If the magnetic force exceeds the inward force, the particles stop their circulation around the vortex core, consequently breaking the structure. This problem can be solved because the vortex inward force is proportional to the moving velocity. Therefore, by simply increasing the rotating frequency of the magnetic field to reach the step-out frequency, the vortex can maintain its structure while performing the translation motion with the applied gradient field. If the rotational frequency is further increased beyond the step-out frequency, the vortex starts to elongate the structure and splits into smaller vortices.

Figure S4 (d) illustrates the successful generation of vortex-like swarming and its locomotion using the proposed approach. By applying a magnetic field (20 mT) and rotating frequency (20 Hz), multiple micro-vortices were quickly generated and merged into a larger vortex (Figure S4(d)) because they approached the critical distance (a/d–0.3) ^9^. At t = 44 s, the merging of two vortices can be observed, and the final vortex forms at t = 50 s with most of the particles gathered in the vortex core. The remaining particles tend to gather locally and merge with the large vortex. Because of the high inward force with a high rotating frequency, the vortex maintains its morphology and synchronizes its motion with the application of a 200 mT/m gradient field, as shown in Figure S4(d) at t = 61 s. The step-out frequency and maximum gradient that can be used while maintaining the vortex depend on the magnetic field strength. The higher the applied magnetic field, the higher the step-out frequency; hence, a higher magnetic gradient can be used to move the vortex due to the high inward force. The maximum step-out frequency and maximum gradient field used in this work were experimentally determined; these were 20 Hz and 150 mT/m at 8 mT and 40 Hz and 400 mT/m at 20 mT, respectively. In Figure S4(c), the combined alternative uniform field and field gradient (20 mT and 200 mT/m, respectively) are applied perpendicular to the surface at a frequency 10 Hz. Within 10 s, the vortex-like swarm and particle chains are quickly broken up (Figure S4(e)).

**References**

1. Go G, Han J, Zhen J, et al. A Magnetically Actuated Microscaffold Containing Mesenchymal Stem Cells for Articular Cartilage Repair. *Adv Healthc Mater*. 2017;6(13):1-10. doi:10.1002/adhm.201601378

2. Petruska AJ, Nelson BJ. Minimum Bounds on the Number of Electromagnets Required for Remote Magnetic Manipulation. *IEEE Trans Robot*. 2015;31(3):714-722. doi:10.1109/TRO.2015.2424051

3. Son D, Dogan MD, Sitti M. Magnetically actuated soft capsule endoscope for fine-needle aspiration biopsy. *2017 IEEE Int Conf Robot Autom*. Published online 2017:1132-1139. doi:10.1109/ICRA.2017.7989135

4. Pourkand A, Abbott JJ. A Critical Analysis of Eight-Electromagnet Manipulation Systems: The Role of Electromagnet Configuration on Strength, Isotropy, and Access. *IEEE Robot Autom Lett*. 2018;3(4):2957-2962. doi:10.1109/LRA.2018.2846800

5. Kummer MP, Abbott JJ, Kratochvil BE, Borer R, Sengul A, Nelson BJ. Octomag: An electromagnetic system for 5-DOF wireless micromanipulation. *IEEE Trans Robot*. 2010;26(6):1006-1017. doi:10.1109/TRO.2010.2073030

6. Kratochvil BE, Kummer MP, Erni S, et al. MiniMag: a hemispherical electromagnetic system for 5-DOF wireless micromanipulation. In: *Experimental Robotics*. ; 2014:317-329.

7. Lecuona A, Ruiz-Rivas U, Nogueira J. Simulation of particle trajectories in a vortex-induced flow: Application to seed-dependent flow measurement techniques. *Meas Sci Technol*. 2002;13(7):1020-1028. doi:10.1088/0957-0233/13/7/308

8. Ash RL, Khorrami MR. *Vortex Stability*.; 1995. doi:10.1007/978-94-011-0249-0

9. Saffman PG, Szeto R. Equilibrium shapes of a pair of equal uniform vortices. *Phys Fluids*. 1980;23(12):2339-2342. doi:10.1063/1.862935

Supplementary Tables

**Table S1.** **Design parameters of the EMA system.**

| **Parameter** | **Value** |
| --- | --- |
| Workspace diameter (mm) | 40 |
| Distance from end of coil  to center of workspace (mm) | 60 |
| Wire diameter (mm) | 1.6 |
| Number of turns | 1368 |
| Coil inner diameter (mm) | 40 |
| Coil outer diameter (mm) | 77 |
| Coil length (mm) | 210 |
| Core diameter (mm) | 40 |
| Core length (mm) | 240 |
| Resistance (Ω) | 2.2 |

**Table S2.** **Angle range of coils within the region of coil placement (Figure S1).**

| **Region of coil placement** | **Upper region** | **Lower region** |
| --- | --- | --- |
| Angle range of coil placement  (Deg.) | *θ:* 45°, 135°, 225°, 315°  *α:* 0°$\leq$*α*$\leq$-10° | *θ:* 0°$\leq$*θ*$<$360°  *α:* -40°$\leq$*α*$<$-50° |

**Table S3. Results of optimized coil configuration (Figure S2).** *min* indicates a minimum.

| **Candidate** | | **Optimal coil configuration** | | | | | | | | |
| --- | --- | --- | --- | --- | --- | --- | --- | --- | --- | --- |
| **Coil number** | | **1** | **2** | **3** | **4** | **5** | **6** | **7** | **8** | **9** |
| Coil placement  (Deg.) | *θ* | 45 | 135 | 225 | 315 | 0 | 72 | 144 | 216 | 288 |
|  | *α* | 0 | 0 | 0 | 0 | -45 | -45 | -45 | -45 | -45 |
| $\text{(}\prod_{i=1}^{6} \sigma_{i})min$ | | 9.434e–23 | | | | | | | | |
| $\text{(}\sigma_{6}/\sigma_{1})min$ | | 0.0147 | | | | | | | | |
| $\sigma_{6,min}$ | | 1.870e–3 | | | | | | | | |

**Table S4.** **Specification of EMA system with optimal coil configuration.** $\boldsymbol{H}_{simulated}$ and $\boldsymbol{H}_{measured}$ are simulated and measured magnetic field intensities at the center of workspace for 1A of each coil. $\frac{\left| \boldsymbol{H}_{\mathrm{simulated}} \right|}{\left| \boldsymbol{H}_{\mathrm{measured}} \right|}$ is amplitude error between simulated and measured magnetic field intensities. *Error_θ* and *Error_α* are the angular errors between the simulated and measured magnetic field intensities represented by the polar and azimuthal angles (*θ* and *α*) of the spherical coordinate system.

| ***Coil number*** | **Coil 1** | **Coil 2** | **Coil 3** | **Coil 4** | **Coil 5** | **Coil 6** | **Coil 7** | **Coil 8** | **Coil 9** |
| --- | --- | --- | --- | --- | --- | --- | --- | --- | --- |
| ***H_simulated_***  ***(A/m)*** | -1541.5 | 1534.3 | 1533.4 | -1541.8 | -1340.4 | -380.63 | 1043.1 | 1043.4 | -379.69 |
|  | -1538.7 | -1535.2 | 1533.7 | 1540.2 | 0.3813 | -1260.7 | -736.57 | 737.3 | 1259.7 |
|  | -500.57 | -495.75 | -494.76 | -499.61 | 1080.2 | 1073 | 1058.2 | 1058.2 | 1072.3 |
| ***H_measured_***  ***(A/m)*** | -1526.7 | 1526.7 | 1576.7 | -1673 | -1416.7 | -363 | 1023 | 1066.7 | -320 |
|  | -1506.7 | -1580 | 1576.7 | 1553 | 6.7 | -1180 | -760 | 753.3 | 1180 |
|  | -470 | -443.3 | -500 | -583 | 1186.7 | 1010 | 1013 | 953.3 | 940 |
| $\frac{\left\vert\boldsymbol{H}_{\boldsymbol{simulated}} \right\vert}{\left\vert\boldsymbol{H}_{\boldsymbol{measured}} \right\vert}$ | 1.02 | 0.99 | 0.97 | 0.95 | 0.93 | 1.06 | 1.02 | 1.03 | 1.1 |
| ***Error_θ (^o^)*** | 0.3 | -1 | 0 | 2.1 | -0.3 | 0.3 | -1.4 | 0 | -1.6 |
| ***Error_α (^o^)*** | 0.6 | 1.5 | 0.2 | -1.4 | -1.1 | -0.1 | 1.2 | 3.5 | 1.6 |

Supplementary Figures


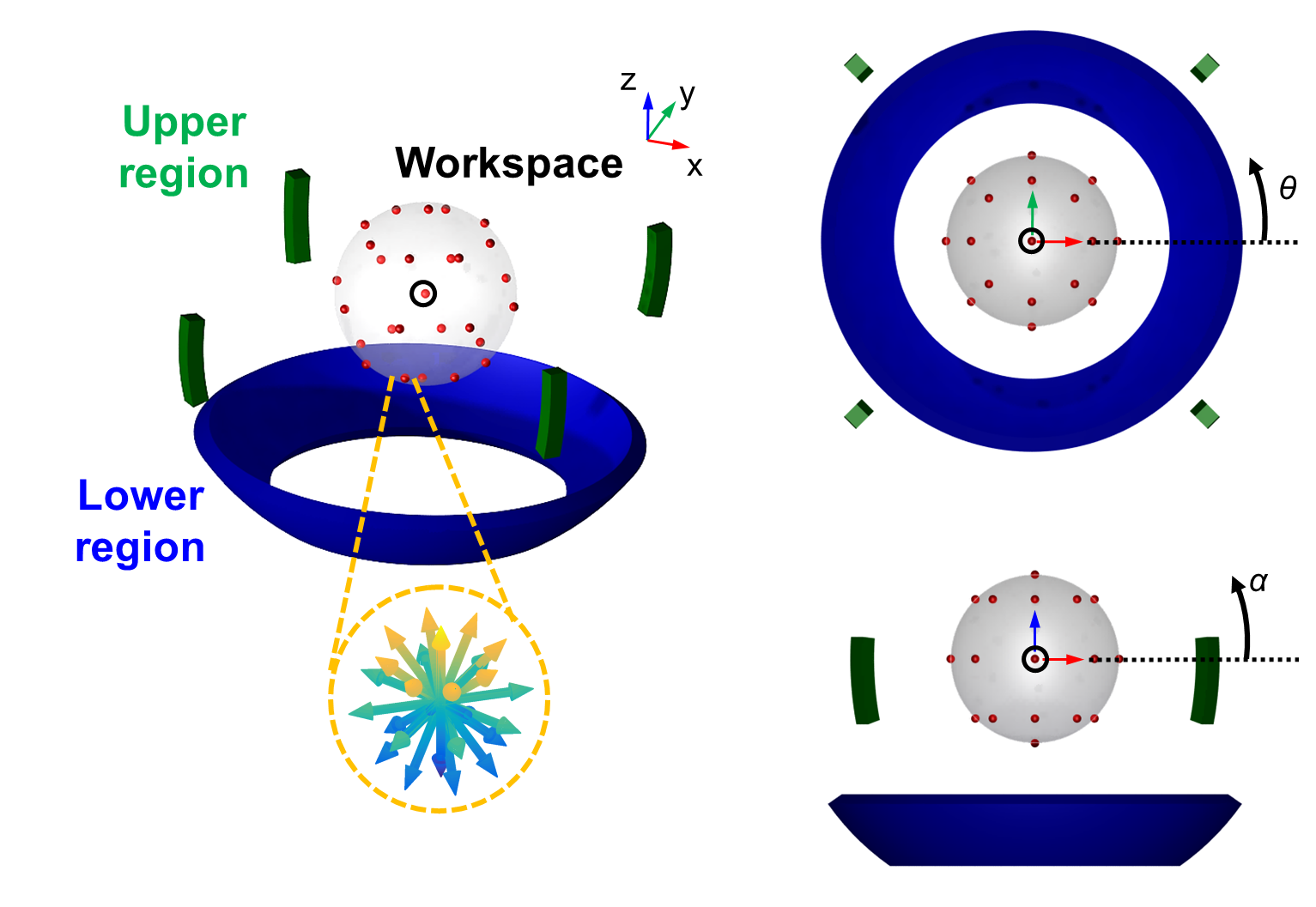


**Figure S1.** Upper and lower regions of coil placement. The axes of all coils point to the center of workspace indicated by a black circle. θ and α represent the polar and azimuthal angles, respectively, of the coil axis in spherical coordinates relative to the center of the workspace. Twenty-seven red points indicate positions of microrobots within the workspace. The yellow dashed circle shows that each point considers 26 orientations of the microrobot.


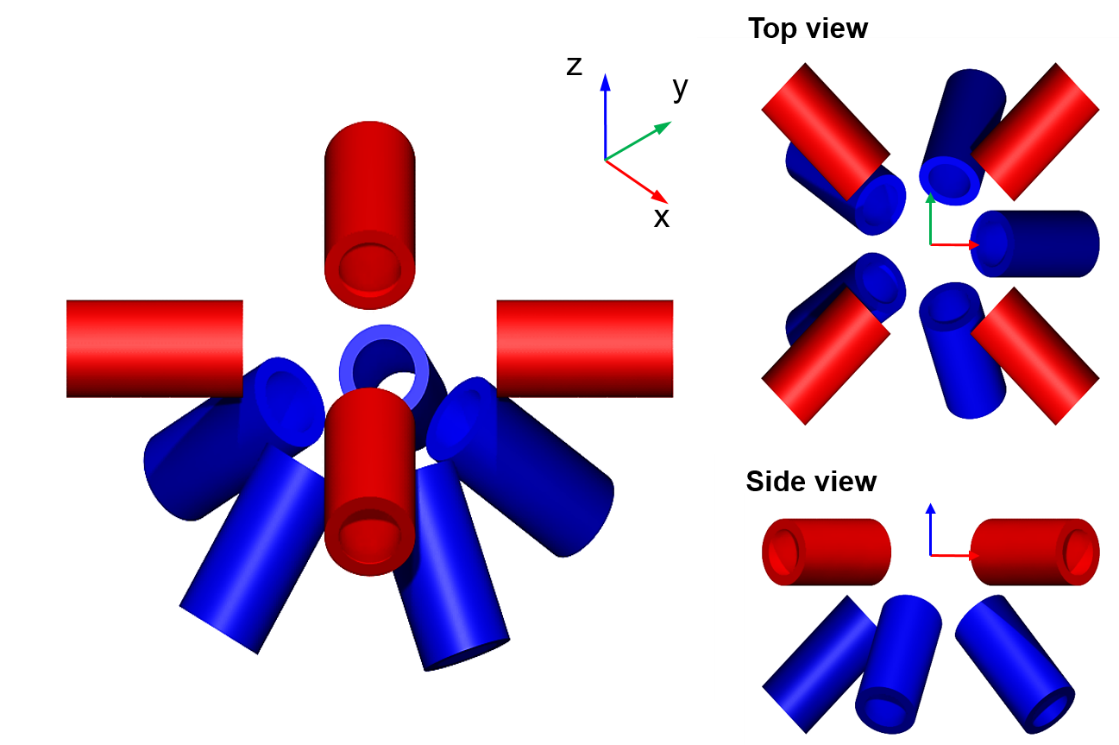


**Figure S2.** A coil configuration among coil configuration candidates obtained through the optimization routine. Detailed parameters of each coil configuration are presented in Table S1.

**
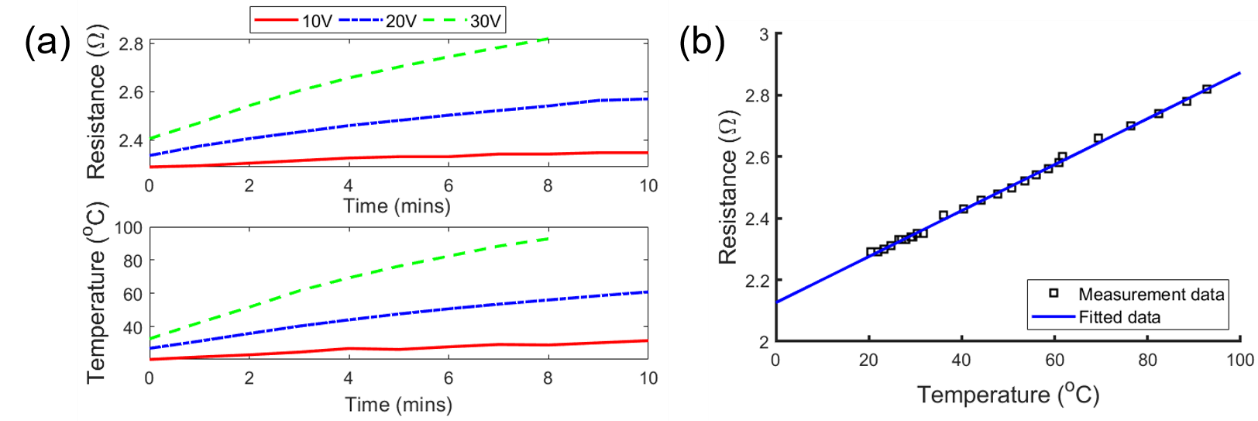
**

**Figure S3.** Measurement data of coil temperature and calculated resistance with respect to the applied voltage in time domain (a). The heating rate of the coil is 1.5^o^C/min, 4^o^C/min, and 9.7^o^C/min under applied voltage of 10, 20, and 30V, respectively. (b) Fitting of coil’s resistance against its temperature where the resistance increasing rate, *dR,* is about 0.007472 Ω/^o^C

**
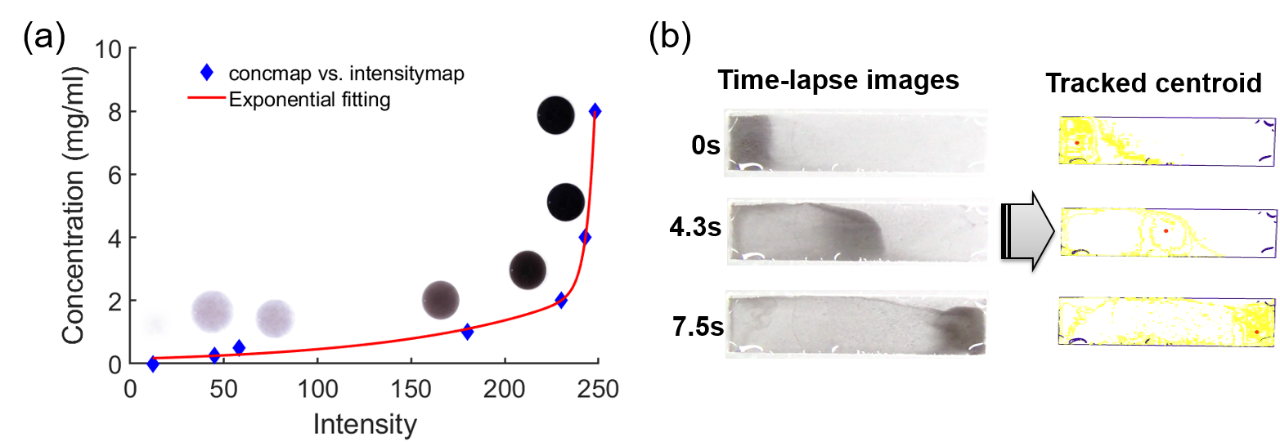
**

**Figure S4.** Calibration of particle’s concentration and image intensity for tracking the centroid of the nanorobot cluster and velocity measurement (a) Time-lapse images and chain-liked cluster centroid tracking in the velocity test. (b).

**
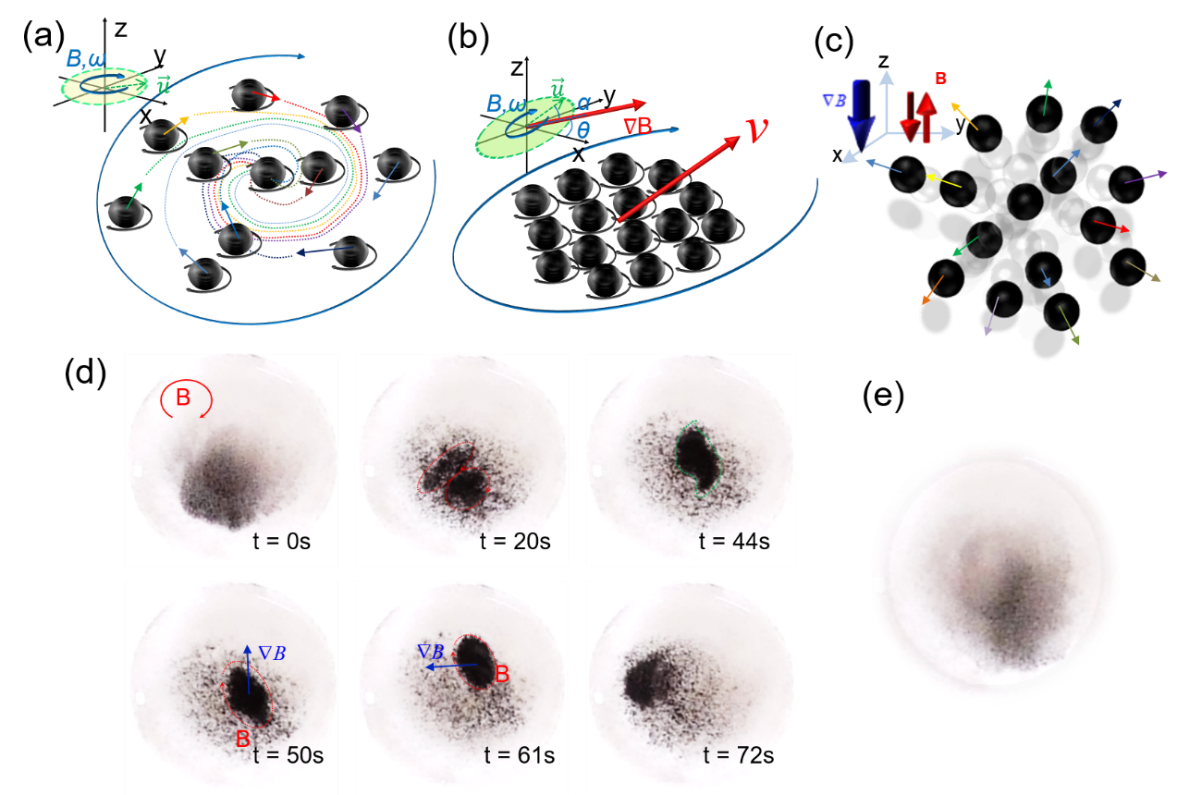
**

**Figure S5.** **Swarm motion vortex-like structure utilizing gradient field.** (a)-(b) 3-D schematic of field and gradient field applied to generate and control the locomotion of the vortex-like swarming motion. (c) Disaggregation mechanism of particle chain. A combination of perpendicular alternative field and gradient field breaks the structure by chain-chain and dipole-dipole repulsion which illustrated in capture image (e). (d) The captured images of the nanoparticles during generation and control the direction of the vortex-like swarming motion.


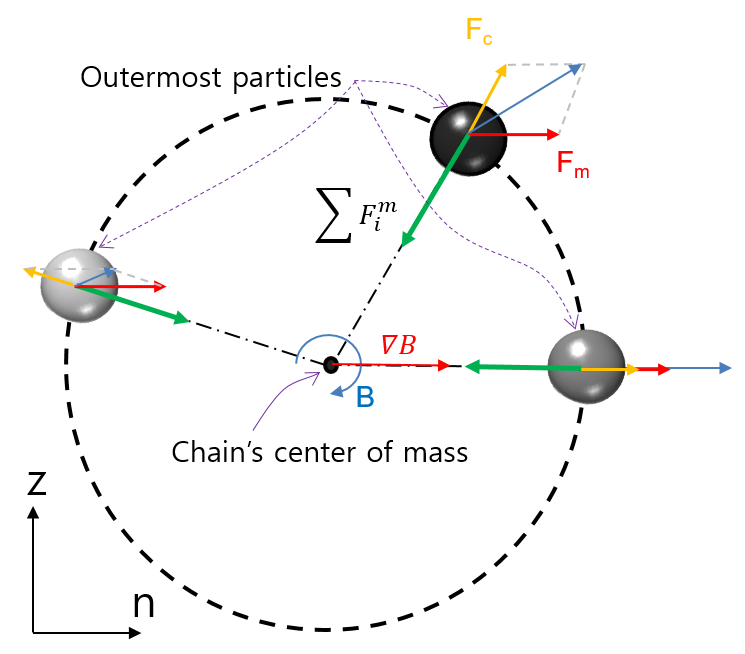


**Figure S6**. Schematic of force distribution during the enhanced tumbling motion under rotating field and directional gradient field The orange arrow represents the centrifugal force, Fc, on the outermost particles in the chain-liked cluster which is proportion to the rotating frequency. The red arrow illustrates the magnetic force exerted on a single particle, contributed to the change in size of magnetic chains, especially on the outermost particles which is always in horizontal direction. The green arrow is the dipole-dipole interaction force of outermost particle’s neighbors which mostly head to chain’s center of mass.


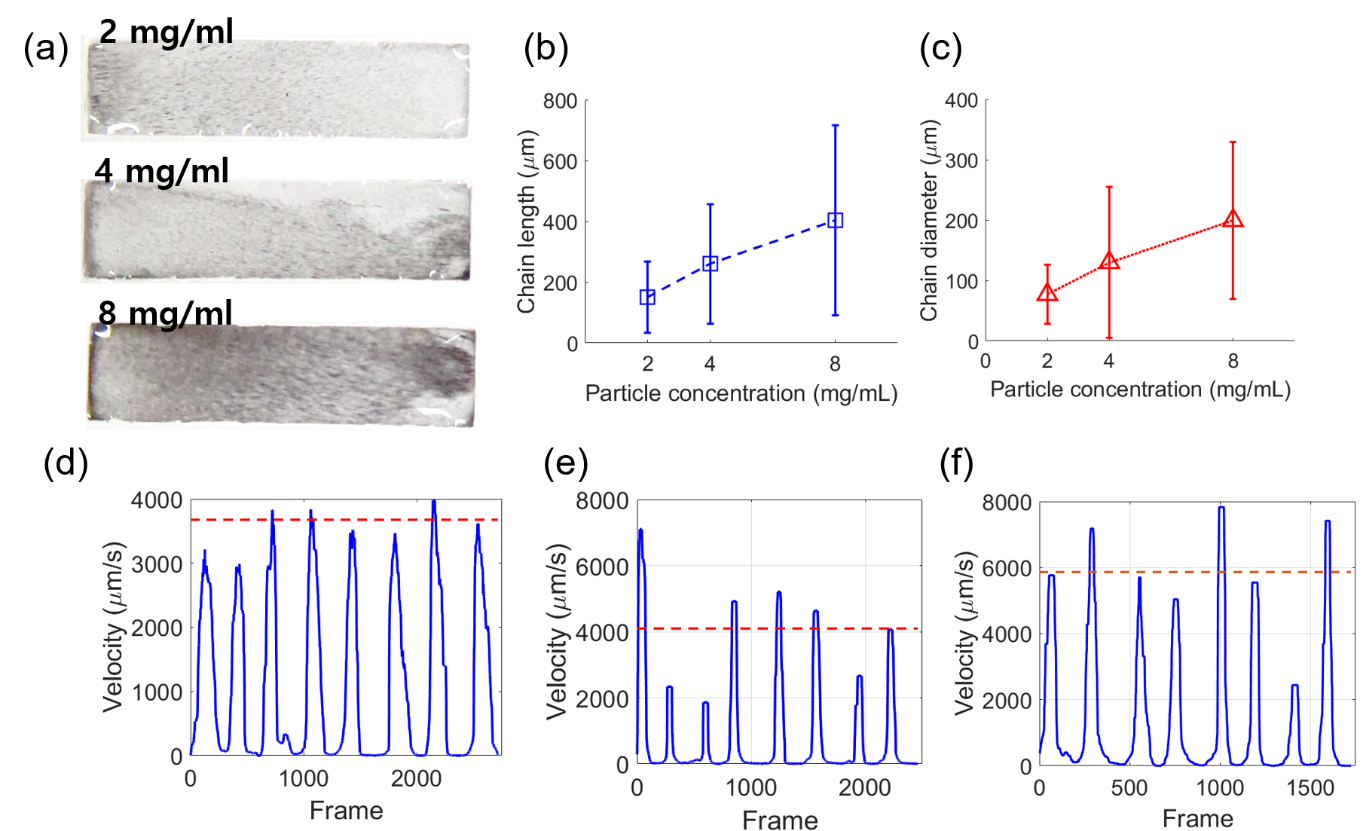


**Figure S7.** (a) Captured images of nanoparticles inside a PBS channel with different particle’s concentration of 2, 4, 8 mg/mL. (b)-(c) Estimated lengths and diameters of the chains under enhanced tumbling motion with various of particle’s concentrations. (d), (e), and (f) are the tracked moving speed of the center of clusters with particle particle’s concentration of 2, 4, and 8 mg/mL, respectively.


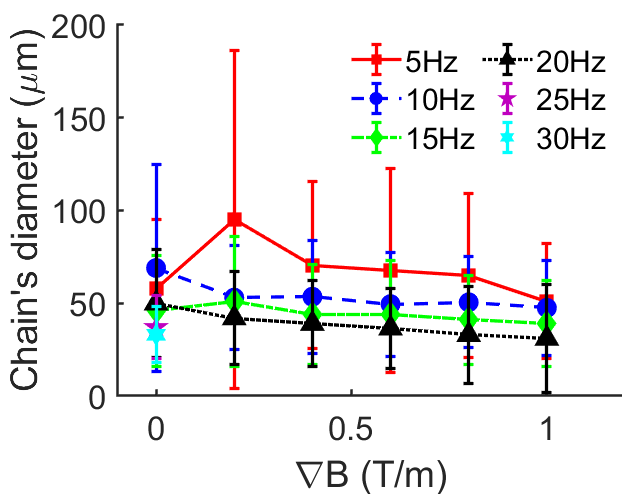


**Figure S8.** Dependence of chain’s diameter to the applied rotating field (5, 10, 15, 20, 30 Hz) and gradient field (0, 0.2, 0.4, 0.6, 0.8, 1 T/m) with constant field’s strength of 10 mT.


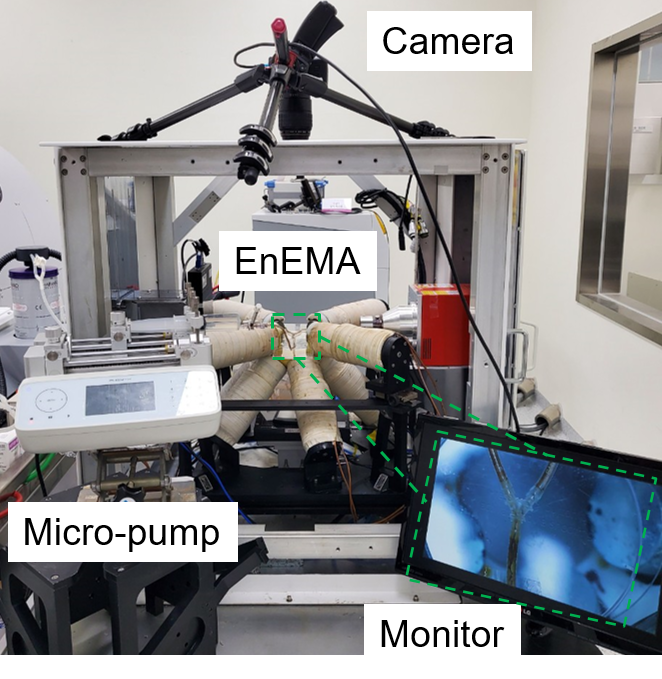


**Figure S9.** The experimental setup for the targeting test with flow. A “Y” fluidic channel made of polymer tube and T-tube connector. The micro-pump is used to control the flow rate. All of targeting performances were recorded by the DSLR camera.
